# Gut microbiota promotes macrophage M1 polarization in hepatic sinusoidal obstruction syndrome via regulating intestinal barrier function mediated by butyrate

**DOI:** 10.1080/19490976.2024.2377567

**Published:** 2024-07-16

**Authors:** Si Zhao, Han Zhang, Hanlong Zhu, Tianming Zhao, Jingjing Tu, Xiaochun Yin, Suzhen Yang, Wei Zhang, Feng Zhang, Ming Zhang, Bing Xu, Yuzheng Zhuge, Jiangqiang Xiao

**Affiliations:** a Department of Gastroenterology, Nanjing Drum Tower Hospital, Affiliated Hospital of Medical School, Nanjing University, Nanjing, Jiangsu, China; b Department of Gastroenterology and Hepatology, Jinling Hospital, Affiliated Hospital of Medical School, Nanjing University, Nanjing, Jiangsu, China; c Department of Gastroenterology, Nanjing Drum Tower Hospital, Chinese Academy of Medical Science & Peking Union Medical College, Nanjing, Jiangsu, China; d Department of Gastroenterology, Nanjing Zhongda Hospital, Nanjing, Jiangsu, China

**Keywords:** HSOS, gut–liver axis, gut microbiota, M1 macrophage, butyrate, intestinal barrier

## Abstract

**Background:**

The intestinal-liver axis is associated with various liver diseases. Here, we verified the role of the gut microbiota and macrophage activation in the progression of pyrrolizidine alkaloids-induced hepatic sinusoidal obstruction syndrome (PA-HSOS), and explored the possible mechanisms and new treatment options.

**Methods:**

The HSOS murine model was induced by gavage of monocrotaline (MCT). An analysis of 16S ribosomal DNA (16S rDNA) of the feces was conducted to determine the composition of the fecal microbiota. Macrophage clearance, fecal microbiota transplantation (FMT), and butyrate supplementation experiments were used to assess the role of intestinal flora, gut barrier, and macrophage activation and to explore the relationships among these three variables.

**Results:**

Activated macrophages and low microflora diversity were observed in HSOS patients and murine models. Depletion of macrophages attenuated inflammatory reactions and apoptosis in the mouse liver. Moreover, compared with control-FMT mice, the exacerbation of severe liver injury was detected in HSOS-FMT mice. Specifically, butyrate fecal concentrations were significantly reduced in HSOS mice, and administration of butyrate could partially alleviated liver damage and improved the intestinal barrier in vitro and in vivo. Furthermore, elevated lipopolysaccharides in the portal vein and high proportions of M1 macrophages in the liver were also detected in HSOS-FMT mice and mice without butyrate treatment, which resulted in severe inflammatory responses and further accelerated HSOS progression.

**Conclusions:**

These results suggested that the gut microbiota exacerbated HSOS progression by regulating macrophage M1 polarization via altered intestinal barrier function mediated by butyrate. Our study has identified new strategies for the clinical treatment of HSOS.

## Introduction

Hepatic sinusoidal obstruction syndrome (HSOS), initially described as hepatic venous occlusive disease (HVOD), is a rare disease targeting hepatic sinusoids, sublobular veins and central veins of the hepatic lobules, which results in destructive damage to hepatic sinusoidal endothelial cells (SECs), congestion of hepatic sinusoids, and intrahepatic portal hypertension. In China, 50%-89% of reported cases of HSOS result from the ingestion of pyrrolizidine alkaloid (PA)-containing products, differing from those in Western countries, which are dominated by hematopoietic stem cell transplantation (HSCT)-related HSOS or oxaliplatin-induced HSOS.^1,2^ It is currently believed that PA-induced hepatotoxicity involves the formation of pyrrole-protein adducts and the depletion of reduced glutathione (GSH) to destroy hepatic sinusoidal endothelial cells, which are considered the primary mechanisms caused by active metabolites of PAs.^3^ Several studies have focused on the direct liver damage initiated by PAs, particularly for cellular injury such as oxidative stress, DNA damage, apoptosis and proinflammatory responses.^4–8^ Drug interventions including exogenous antioxidants, antiapoptotic agents, and anticoagulation therapy have also been studied and applied to some extent.^9^ However, the treatment effect on PA-HSOS is not high, and approximately 50% of patients eventually die due to liver failure. With the proposal of the Nanjing consensus, anticoagulation-transjugular intrahepatic portosystemic shunt (TIPS) stepwise therapy has greatly improved the outcomes of patients.^10^ The existing theory of a direct drug damage mechanism cannot explain the efficacy of anticoagulation and TIPS. In addition, a significant portion of patients choose medication treatment as much as possible due to concerns about surgical risks. Thus, novel treatment avenues may be derived from a better understanding of the PA-HSOS progression molecular mechanism.

The main causes of death in PA-HSOS patients were liver failure and multiple organ injury.^9^ Like other causes of liver failure, many inflammatory factors are found in PA-HSOS patients, such as IL-6, TNF-α, and IL-1β.^5^ Studies have demonstrated that liver macrophages are essential to sustain tissue homeostasis.^11,12^ Macrophages belonging to the innate immune system exhibit a high degree of plasticity and can adapt their phenotype in response to environmental stimuli. In particular, M1 macrophages play a major role in secreting proinflammatory cytokines and producing reactive oxygen species (ROS), which can trigger a systemic inflammatory response, ultimately resulting in liver failure and even multiple organ failure.^13^ It has been well reported that macrophage-regulated processes are implicated in liver disease progression, including nonalcoholic fatty liver disease (NAFLD), liver cirrhosis, hepatocellular carcinoma (HCC) and others.^1,14–16^ These studies suggest that the activation of macrophages parallels disease activity, severity, or therapeutic responsiveness by modulating the inflammatory immune response and shaping the inflammatory microenvironment.

In recent years, interactions between the gut microbiota and liver diseases have attracted considerable attention. Previous literature has indicated that macrophage activation can be mediated by a variety of factors, such as bacterial translocation (BT) and endotoxemia, due to increased gut permeability, as well as altered composition in the intestinal microbiota and its metabolites, including certain cholesterol and free fatty acids.^17^ Moreover, Lin et al. first demonstrated the existence of intestinal injury in the PA-HSOS animal model, further resulting in progressive hepatic damage, which suggested an important role for the gut – liver axis in the progression of PA-HSOS.^18^ With in-depth study, the relationship between the intestinal microflora and PA-HSOS has gradually been reported. Evidence has revealed that the microbiota in PA-HSOS animals is different from that of healthy individuals, and the abundance of potentially pathogenic bacteria is significantly increased.^19,20^ However, the involvement of the gut bacterial flora in the development of PA-HSOS has not been fully elucidated, particularly short-chain fatty acids (SCFAs), which are thought to mediate the health benefits of the intestinal microbiome. In this study, we observed gut microbiota dysbiosis and impairment of the gut barrier in PA-HSOS patients and mice and that gut microbiota dysbiosis further resulted in enhanced M1 macrophage polarization via alteration of intestinal barrier function mediated by butyrate, aggravating the progression of PA-HSOS.

## Materials and methods

### Patient recruitment and specimens

Pathologically confirmed PA-HSOS paraffin-embedded liver and colon tissues with corresponding normal tissues were obtained from the Department of Pathology, Drum Tower Hospital Medical School of Nanjing University. Stool and peripheral blood samples were also collected from PA-HSOS patients and healthy volunteer donors. The characteristics of the participants were summarized in Supplementary Table S1. The exclusion criteria for stool collection were as follows: antibiotic intake within the last three months, constipation or diarrhea, severe gastrointestinal disease, and combined with tumors. Informed consent was obtained from all participants and the study was performed in accordance with the principles outlined in the Declaration of Helsinki and approved by the Nanjing Drum Hospital Clinical Research Ethics Committee (Ethical approval code: 2021-409-01).

#### Animal experiments

Animal experiments were approved by the Animal Ethics Committee of Nanjing Drum Tower Hospital (Ethical approval code: 2021120003). Eight-week-old male C57BL/6 wild-type (WT) mice (20-22 g) were purchased from Nanjing Qingzilan Technology Co. Ltd (Jiangsu, China). Throughout the experimental periods, animals were maintained in a specific pathogen-free (SPF) environment at 22°C with 12 hour/12 hour cycles of darkness and light. Mice fasted for 12 h and were then gavaged with 750 mg/kg MCT (Sigma-Aldrich, St. Louis, MO, USA) or sterile water to establish a murine model of PA-induced HSOS at different fumic, which was a well-established reproducible model of HSOS.^21^ MCT was first solubilized in physiological saline with 0.1N hydrochloric acid until completely dissolved, then neutralized with 0.1N sodium hydroxide solution (NaOH) up to a pH = 7.0. The solution was protected from light and used immediately after it was ready. For the liver macrophages depletion, mice were injected intraperitoneally with 200uL of clodronate liposomes (FormuMax, USA) 24 h before receiving MCT gavage. In the experiment with antibiotic cocktail (ABX) treatment, mice were administered intragastric ampicillin (200 mg/kg), metronidazole (200 mg/kg), neomycin sulfate (200 mg/kg), and vancomycin hydrochloride (100 mg/kg) once daily for 5 days.^22,23^ All antibiotics were purchased from Sigma-Aldrich, USA. To supplement with sodium butyrate (NaB), we established a relatively chronic HSOS model (one week) with a dosage of 400 mg/kg given twice a week, considering the long-term effectiveness of butyrate. Mice received 200 mM freshly prepared NaB (Beyotime, China) dissolved in drinking water every day and MCT at the same time until the mice were sacrificed.^24^ The solution was replaced every 3 days. After one week of MCT gavage, all mice were sacrificed and liver, colon, feces, and blood were collected for subsequent analyses. In this study, euthanasia was carried out using carbon dioxide inhalation in accordance with the American Veterinary Medical Association (AVMA) guidelines for euthanasia of animals. Moreover, humane endpoints were performed when weight loss exceeded 20% or inability to freely eat or drink for 24 h.

#### Fecal microbiota transplantation (FMT)

We first prepared fecal hydration liquid as described previously.^25^ Briefly, feces (150 mg) from WT and MCT-induced mice were weighed and diluted in 1 mL of sterile saline solution, shaken vigorously for 3 min followed by filtration with a 40 μm filter sieve. After that, a 10% glycerol solution was added to the filtrate and stored at − 80°C until undergoing FMT. The mice were divided into two groups: the CTRL-FMT and the HSOS-FMT group. The detailed operations were as follows. All FMT recipient mice were pretreated with antibiotics (as indicated above) for five consecutive days and then orally gavaged with 250 μl of the fecal hydration liquid daily for 8 days. The CTRL-FMT mice were gavaged with the fecal hydration liquid from healthy mice, while the HSOS-FMT mice were gavaged with the fecal hydration liquid from the HSOS mice. After the final fecal liquid administration, all mice were gavaged with MCT. To maintain the efficacy of FMT, the fecal suspensions were sub-packed to avoid repeated freezing, and we ensured that one single mouse donor corresponded to one recipient.

### Cell culture and reagents

Human normal liver cell LO2 and human intestinal epithelial cell NCM460 (Pricella Life Science&Technology Co., Ltd) were cultured in RPMI medium 1640 (BioChannel) supplemented with 10% fetal bovine serum (BioChannel) and 1% (V/V) penicillin/streptomycin. The cells were maintained at 37°C in 5% CO2 atmosphere. In the fecal supernatant treatment experiment, supernatant was extracted from patients with PA-HSOS and healthy subjects following the method described in previously published literature.^26,27^ Subsequently, the NCM460 cells were treated with 10% HSOS supernatant for 24 h, while the control groups were treated with 10% healthy subjects supernatant or PBS for 24 h. In order to explore the role of NaB in LO2 and NCM460 cells, butyrate was added at different concentrations (1-5 mM) for 24 h.

#### Isolation of nonparenchymal cells (NPCs) in the liver

The isolation of NPCs is referred to Chen et al. with some modifications.^28^ In brief, the mice were anesthetized by pentobarbital sodium injections to achieve deep anesthesia and perfused via the portal vein with calcium-free hanks solution for 6 minutes until blood was removed, followed by 0.03% collagenase digestion (Type IV, Sigma). Single cell suspensions were isolated from the liver by using forceps, filtered through a 70 µm cell strainer, and subsequently centrifuged at 50 g for 5 min. NPCs in the supernatant were harvested and further centrifuged. Purification of NPCs was continued using a Percoll density gradient (Solarbio, Beijing, China). Red blood cells were removed using erythrocyte lysate (Pharm Lyse, BD Biosciences). Prepared cell suspensions were used for flow cytometric analysis.

#### Flow cytometry

For flow staining of mouse liver NPCs, the cells were resuspended in the precooling cell staining buffer (BD), blocked by Fc-receptor block (BD), and then stained with anti-mouse PE-Cy7-conjugated CD45, anti-human/mouse FITC-conjugated CD11b, and anti-mouse PE-conjugated F4/80. All surface staining antibodies above were bought from BioLegend. Cell apoptosis assays were conducted using Annexin V-fluorescein isothiocyanate (FITC) apoptosis detection kit (Keygen BioTECH) according to the manufacturer’s instructions. Analysis of stained cells was performed using BD FACS Calibur flow cytometers and FlowJo software.

### Histopathological analysis and immunohistochemistry

A slice of mice liver and specimens from the colon were taken and fixed in 4% paraformaldehyde for 24 h, followed by paraffin embedding to prepare 5um thick sections. Overall morphology was assessed by hematoxylin-eosin (HE) staining, and Alcian blue-Periodic acid Schiff (AB-PAS) was evaluated for the amount of mucus secretion. Liver tissue sections from patients were stained for CD68 (1:1000, Abcam, ab955). Macrophages and granulocytes were identified by immunostaining with mouse F4/80 (1:100, Abcam, ab111101) and Ly6G (1:1000, Abcam, ab238132), respectively. For antigen repair, we used citrate buffer (pH = 6.0) in a microwave oven for 15 minutes.

### Immunofluorescence

NCM460 cells with different reagents were seeded into 24-well plates for cell climbing. Afterward, the cells were fixed in 4% paraformaldehyde at room temperature for 15 minutes. After washing and blocking with 10% sheep serum for 1 h, the cells were incubated overnight with ZO-1 (1:200, Proteintech, 21,773–1-AP) and Occludin (1:400, Proteintech, 27,260–1-AP) at 4°C. The following day, the cells were stained with corresponding secondary antibodies at room temperature for 1 h, sealed with DAPI-containing medium, and observed under a fluorescent microscope. A single- or double-color immunofluorescence staining in tissue sections was performed as previously described. The following primary antibodies were used: anti-iNOS (1:100, Immunoway, YT3169), anti-CD206 (1:200, CST, 24,595), anti-Ly6G (1:100, Abcam, ab238132), ZO-1 (1:100, Proteintech, 21773–1-AP), anti-Occludin (1:200, Proteintech, 27,260–1-AP), anti-E-cadherin (1:200, CST, 14,472). Secondary antibodies were Alexa Fluor 488 goat anti-mouse IgG (1:1000, Abcam), Alexa Fluor 488 goat anti-rabbit IgG (1:1000, Abcam) and Alexa Fluor 568 goat anti-rabbit IgG (1:1000, Abcam).

### Western blot analysis

Protocols for protein isolation and detection of protein expression levels have been described previously.^24^ The following dilutions of primary antibodies were used: anti-Bax (1:1000, CST, 14796S), anti-Bcl-2 (1:1000, CST, 3498S), anti-Caspase-3 (1:1000, Abclonal, A21677), anti-iNOS (1:1000, Immunoway, YT3169), anti-IL-1β (1:1000, Immunoway, YT5201), anti-CD163 (1:1000, CST, 68922S), anti-Arg-1 (1:1000, Abclonal, A4923), anti-ZO-1 (1:1000, Santa Cruz, sc-33,725), anti-Occludin (1:1000, Proteintech, 27,260–1-AP), anti-E-cadherin (1:1000, CST, 14,472), anti-β-catenin (1:2000, Proteintech, 51,067–2-AP), anti-β-actin (1:3000, Proteintech, 81,115–1-RR).

#### Quantitative real-time PCR

A protocol provided by the manufacturer was followed for the isolation of total RNA from tissues and cells using TRIzol reagent (TaKaRa, Kusatsu, Japan). LightCycler 96 Fluorescent PCR was used to perform quantitative amplification using SYBR Green as a fluorescent dye. The primers were synthesized by Tsingke Biological (Chongqing, China), and the sequences of the primers were listed in Supplementary Table S2.

#### TUNEL assay

TUNEL staining for liver tissue sections was performed with the TUNEL Apoptosis Detection kit (Roche Applied Science, Mannheim, Germany) to detect necrotic cells following the manufacturer’s instructions. Briefly, sections of liver tissue were dewaxed, then treated with Proteinase K enzyme, followed by 0.1% Triton solution. Subsequently, TdT enzyme, dUTP and buffer at a 1:5:50 ratio were mixed and incubated with tissue at 37°C for two hours. After that, the nuclei were counterstained with DAPI and photographed with a fluorescence microscope.

### Enzyme-linked immunosorbent assay (ELISA)

The levels of LPS in the peripheral blood of patients were measured using a commercial human immunoassay ELISA kit (LiankeBio, Hangzhou, China). The levels of lipopolysaccharides (LPS) in the portal vein blood of mice were quantified using a commercially available mouse ELISA kit (JianglaiBio, Shanghai, China). All procedures were performed in accordance with the manufacturer’s instructions.

### Intestinal permeability detection

In order to measure intestinal permeability in vivo, mice were gavaged with FITC-dextran (4kDa, Sigma, Cat#68059) dissolved in PBS at the dose of 440 mg/kg after 10 h fasting of food and water, and peripheral blood was harvested 4 h later. Serum FITC-dextran concentrations were determined at an excitation wavelength of 490 nm and an emission wavelength of 530 nm by using a fluorescence microplate reader. A standard curve was generated by serially diluting serum FITC-dextran.

### Bacterial fluorescence in situ hybridization (FISH)

Briefly, paraffin-embedded liver sections were subjected to deparaffinization, rehydration, and digestion. After prehybridization at 37°C for 1 h, liver tissues and EUB338 probe (5’-GCTGCCTCCCGTAGGAGT-3’) at a 10 mg/L concentration were hybridized at 37°C overnight. Finally, the slices were washed three times, counterstained with DAPI in the dark, and visualized by epifluorescent microscopy.

### Microbial diversity analysis

The microbial community DNA was extracted using MagPure Stool DNA KF kit B (Magen, China) following the manufacturer’s instructions. DNA was quantified with a Qubit Fluorometer by using Qubit® dsDNA BR Assay kit (Invitrogen, USA) and the quality was checked by running aliquot on 1% agarose gel. Variable regions V4 of bacterial 16S rRNA gene was amplified with degenerate PCR primers, 515F (5’-GTGCCAGCMGCCGCGGTAA-3’) and 806 R (5’- GGACTACHVGGGTWTCTAAT-3’). Both forward and reverse primers were tagged with Illumina adapter, pad and linker sequences. PCR enrichment was performed in a 50 μL reaction containing 30ng template, fusion PCR primer and PCR master mix. PCR cycling conditions were as follows: 95°C for 3 minutes, 30 cycles of 95°C for 45 seconds, 56°C for 45 seconds, 72°C for 45 seconds and final extension for 10 minutes at 72°C for 10 minutes. The PCR products were purified using Agencourt AMPure XP beads and eluted in Elution buffer. Subsequently, the libraries were qualified by the Agilent Technologies 2100 bioanalyzer. The validated libraries were used for sequencing on Illumina HiSeq 2500 platform (BGI, Shenzhen, China) following the standard pipelines of Illumina, and generating 2 × 250 bp paired-end reads.

### SCFAs measurement

Faeces (40 mg per mouse) were supplemented with grinding beads and 50% acetonitrile solution and ground at 60 Hz for 1 min. The samples were then centrifuged at 13000rpm for 10 minutes at 4°C. The supernatant was diluted at an appropriate ratio, and various derivatization reagents were added at 40°C for 30 min. After centrifugation, 20 µl of derivatization solution was added and mixed with 280 µl 50% acetonitrile solution. Following another centrifugation step, pipette out 200 μl of sample and transfer it to a LC-MS vial from the supernatant solution. Additionally, the standards of acetic acid, propionic acid, butyric acid, isobutyric acid, 2-Methylbutyric acid, isovaleric acid, valeric acid, and 3-Methylvaleric acid were also transferred to autosampler vials for further analysis.

### Statistical analysis

Statistical analysis of the data was performed using GraphPad Prism 8 (San Diego, USA). Quantitative data were displayed as the mean±standard deviation (SD), with error bars denoting the SD. The α-diversity of gut microbiota and the abundance of gut microbiota at the phylum and family levels between the two groups were analyzed using Mann-Whitney test. Data of serological indicators, pro-inflammatory cytokines, the proportion of macrophages, tight junction proteins and apoptosis-related markers were analyzed using unpaired t-test (for two groups) or one-way ANOVA (for multiple groups). The correlation between CD68 and serological indicators was conducted by multiple linear regression. A statistically significant difference was considered to be *p* ≤ 0.05. At least three attempts were made to repeat cellular and animal experiments for accuracy. Corresponding t-statistics with *p* values or F-statistics with *p* values were reported in Supplementary Table S3.

## Results

### PA-HSOS patients and mice develop gut microbiota dysbiosis and impaired gut barrier

**Figure 1.**
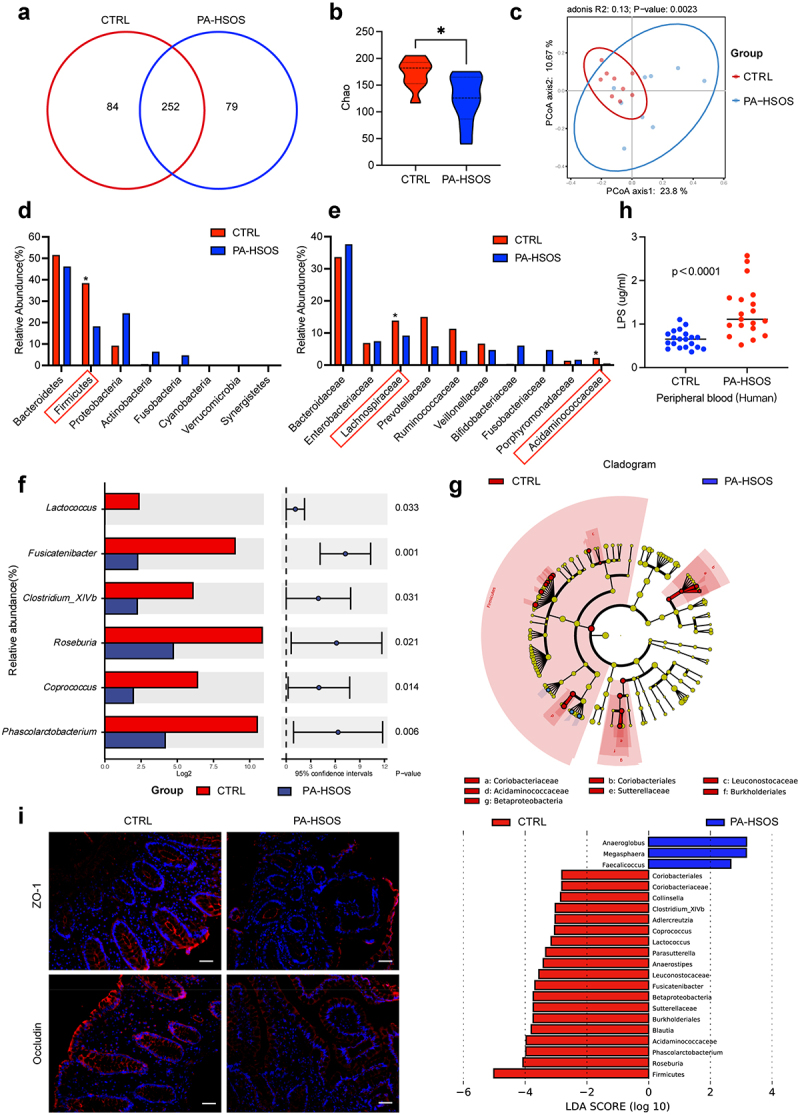
PA-HSOS patients occur gut microbiota dysbiosis and impaired gut barrier. (a) In genus level, venn diagram of the OTUs in the CTRL and PA-HSOS patients (*n* = 9 per group). (b) Chao diversity index of the gut microbiota between the two group. (c) Principal coordinate analysis (PCoA) plot of similarities among the two groups. (d) Bar charts of the gut microbiota composition at the phylum level in CTRL group and HSOS group (top 8). (e) Bar charts of the gut microbiota composition at the family level in CTRL group and HSOS group (top 10). (f) Relative taxon abundance comparison among the HSOS and control groups. Genera with a significance of *p* < 0.05 are partially shown. (g) Cladogram generated from linear discriminant analysis effect size (LEfSe) and the LDA score. (h) The plasma concentrations of LPS in CTRL group (*n* = 20) and PA-HSOS group (*n* = 19). (i) Immunofluorescent staining of ZO-1 and Occludin in the colon sections of patients with control and PA-HSOS. Data are expressed as mean ± SD. Scale bars in images represent 50 μm. ns, no significance, *p < 0.05, **p < 0.01, ***p < 0.001, ****p < 0.0001 as indicated. HSOS, hepatic sinusoidal obstruction syndrome; CTRL, control; MCT, monocrotaline.

We first performed 16S rRNA gene amplicon sequencing to assess the microbiota community structure of the stool samples derived from the control group (*n* = 9) and PA-HSOS patients (*n* = 9). According to the dilution curve method, the depth of the sequencing was sufficient for further analysis (Figure S1A). As shown in Figure 1A, the two groups of samples had 252 similar operational taxonomic unit (OTU) compositions. The Chao, ACE, and Shannon diversity indices were used to evaluate the alpha diversity between the two groups. Most of them exhibited analogous tendencies that HSOS patients manifested significantly reduced alpha diversity compared to the control group (*p* = 0.024, *p* = 0.024, *p* = 0.300 for each index) (Figure 1B and Figure S1B). Based on the PCoA analyses, the structure of the microbial communities differed significantly between the two groups (Figure 1C). The relative abundance of bacteria was also determined to further examine the detailed alterations. At the phylum and family levels, PA-HSOS patients had a significant decrease in the dominant bacteria Firmicutes (38.44% vs. 18.35%, *p* = 0.022) and Lachnospiraceae (16.69% vs. 5.83%, *p* = 0.042) (Figure 1D, E). In particular, as major producers of SCFAs maintaining gut homeostasis, *Phascolarctobacterium* (*p* = 0.006), *Coprococcus* (*p* = 0.014), *Roseburia* (*p* = 0.021), and *Clostridium_XlVb* (*p* = 0.031) were significantly depleted (Figure 1F). Additionally, high-dimensional class comparisons were performed by using linear discriminant analysis (LDA) and effect size analysis (LEfSe) on fecal microbiota composition to identify differentially abundant taxa. As expected, the abovementioned genera exhibited relatively high specificity in healthy controls (*p* < 0.05) (Figure 1G). Additionally, LPS, a biomarker hinting at a damaged intestinal barrier, was drastically elevated in the peripheral blood of PA-HSOS patients compared to healthy subjects (*p* < 0.001) (Figure 1H). Immunofluorescence images showed that tight junction proteins (ZO-1 and Occludin) were significantly inhibited in the colon of PA-HSOS patients (Figure 1I). Similar results were also found in mice (Figure S2A-E and Figure S3A-D). Taken together, these results revealed that PA-HSOS caused dysbacteriosis and obvious intestinal injury.

### Intestinal damage caused by disturbed gut microbiota accelerates the development of PA-HSOS

**Figure 2.**
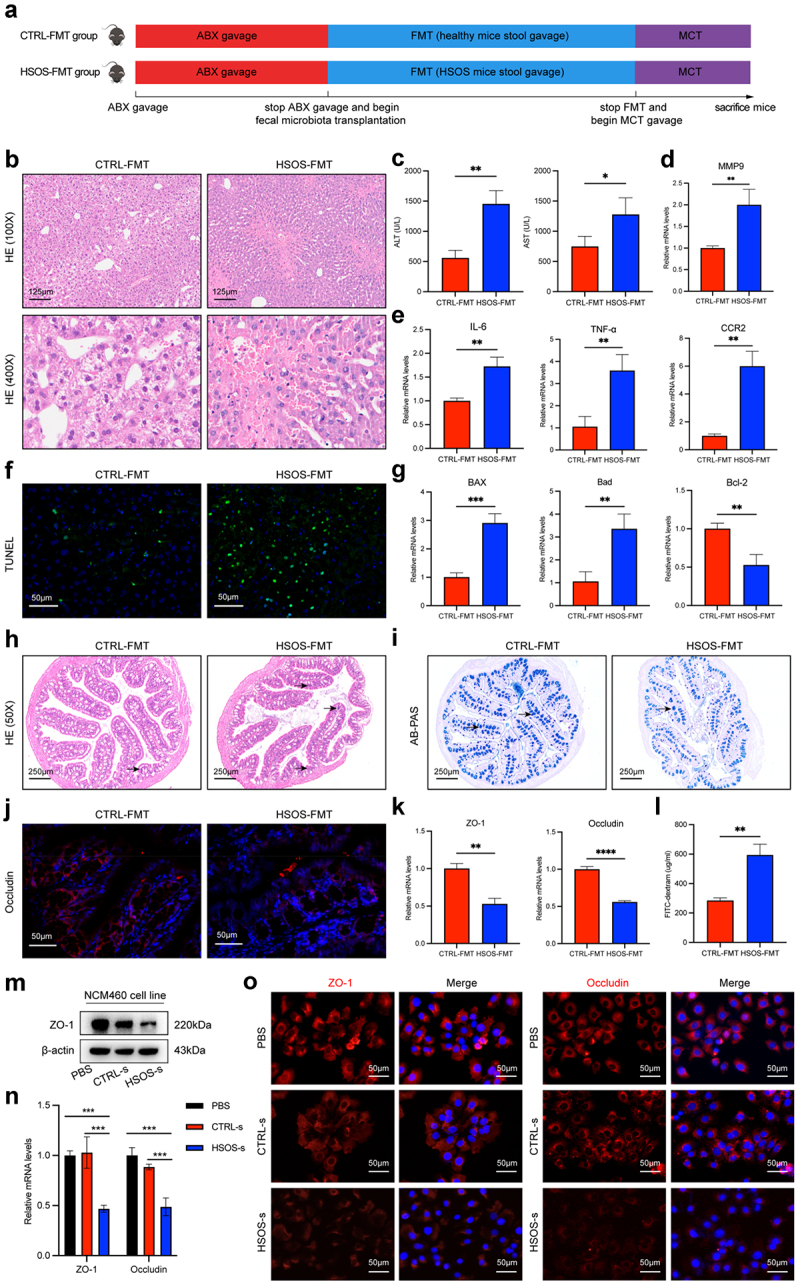
Intestinal damage caused by disturbed gut microbiota accelerates the development of PA-HSOS. (a) Schematic diagram of the mouse HSOS-FMT process. (b) HE histopathology sections of a representative liver in the CTRL-FMT group and HSOS-FMT group. (c) Serum ALT and AST levels in the two groups. (d) The mRNA level of MMP9 in the CTRL-FMT group and HSOS-FMT group. (e) The mRNA expression of proinflammatory factors (IL-6, TNF-α, CCR2) on liver tissue of the two groups. (f) Necrosis induced DNA fragmentation in mouse livers was measured by TUNEL assay in the two groups. (g) RT-qPCR analysis of Bax, Bad, and Bcl-2 on liver tissue of the two groups. (h) Representative images of colon tissues stained by H&E in mice. Black arrow indicated inflammatory cells infiltration. (i) The distribution of goblet cells (black arrow) in colon tissues by AB-PAS staining. (j) Immunofluorescent staining of Occludin in the colon sections in CTRL-FMT and HSOS-FMT mice. (k) The mRNA expression of the ZO-1 and Occludin genes in the colon were detected by RT-qPCR. (l) The serum FITC-Dextran level in mice induced for CTRL-FMT and HSOS-FMT. (m, n) the protein and mRNA levels of ZO-1 and Occludin were evaluated in the NCM460 cells treated with PBS and supernatant of feces from the healthy and HSOS patients for 24 h. (o) Representative immunostaining of ZO-1 and Occludin in NCM460 cells. *N* = 6 per group. Data are expressed as mean ± SD. ns, no significance, *p < 0.05, **p < 0.01, ***p < 0.001, ****p < 0.0001 as indicated. HSOS, hepatic sinusoidal obstruction syndrome; CTRL, control; FMT, fecal microbiota transplantation.

We then performed FMT experiments to gain further insight into the relationship between disordered gut microbes and PA-HSOS. The detailed experimental procedure was shown in Figure 2A. Before performing FMT, we first performed ABX treatment to simulate the relative sterile environment of the gut as previously reported.^22^ At the same time, the clearance efficiency of intestinal flora in the CTRL and ABX mice was evaluated using 16S rRNA sequencing. As shown in Figure S4A, the OTUs were significantly lower in the ABX group (215) than in the control group (1008). There was only a total of 65 similar types of OTUs in the two groups. The alpha (Figure S4B-D) and beta diversity index (Figure S4E-G) demonstrated that ABX group was quite different from the CTRL group in terms of the structural composition of the intestinal microbiota. Cluster analysis disclosed that ABX pretreatment led to a distinct shift at the phylum (Figure S4H), family (Figure S4I), and species (Figure S4J) levels. Moreover, Circos presented that the CTRL group was mainly composed of Firmicutes and Bacteroides, whereas the ABX group mainly consisted of Proteobacteria (Figure S4K). The above results showed that antibiotic cocktail treatment led to a more significant reduction in the bacterial biodiversity and removed most intestinal flora in mice.

Afterward, we transplanted stool from the CTRL and HSOS mice into ABX pretreatment mice and performed MCT gavage. The results showed that augmented liver injury, such as extensive pericentral hepatic necrosis and hemorrhage with simultaneous elevations of alanine aminotransferase (ALT), aspartate aminotransferase (AST), and matrix metalloproteinase 9 (MMP9), was found in HSOS-FMT mice (Figure 2B-D). As increased hepatocyte apoptosis was linked to inflammation and liver injury induced by MCT,^29,30^ hepatic apoptosis was also assessed. CTRL-FMT mice demonstrated smaller areas of liver tissue apoptosis and inflammation than HSOS-FMT mice (Figure 2E-F). Expression of the anti-apoptotic protein Bcl-2 was upregulated and the expressions of pro-apoptotic proteins Bax and Bad were downregulated in the CTRL-FMT mice compared to the HSOS-FMT mice by performing PCR analysis (Figure 2G). Considering that PA-HSOS patients possessed a perturbed alteration of gut microbiota and an increase in intestinal permeability, we hypothesized that the intestinal damage might be partially initiated by gut flora. For hypothesis testing, damage to the intestinal barrier was also assessed by histopathological evaluation. According to HE staining, both groups contained tissue damage and inflammatory cells, but the HSOS-FMT group indicated significantly greater infiltration of inflammatory cells than the CTRL-FMT group (Figure 2H), which was consistent with AB-PAS staining exhibiting reduced numbers of goblet cells (Figure 2I). In addition, the tight junction proteins Occludin and ZO-1 in the HSOS-FMT group decreased compared to those in the control group, as revealed by the results of immunofluorescence and PCR experiments (Figure 2J, K). Moreover, leakage of FITC-dextran was increased in HSOS-FMT mice, but FITC-dextran leakage was blocked in CTRL-FMT mice (Figure 2L). Next, an in vitro model in which NCM460 cells were incubated with the supernatant of feces from healthy (CTRL-s) and PA-HSOS patients (HSOS-s) was also successfully built to explore the effects of gut microbial dysbiosis on intestinal barrier damage. As shown in Figure 2M-O, the levels of ZO-1 and Occludin were dramatically decreased in the HSOS-s group compared with the control groups (PBS group and CTRL-s group). These results demonstrated that HSOS-FMT could exacerbate the development of PA-HSOS dependent on the intestinal barriers, highlighting the essential role of gut microbiota and barrier integrity in regulating the development of disease processes.

### Microbiota-derived butyrate gavage delays PA-HSOS progression

**Figure 3.**
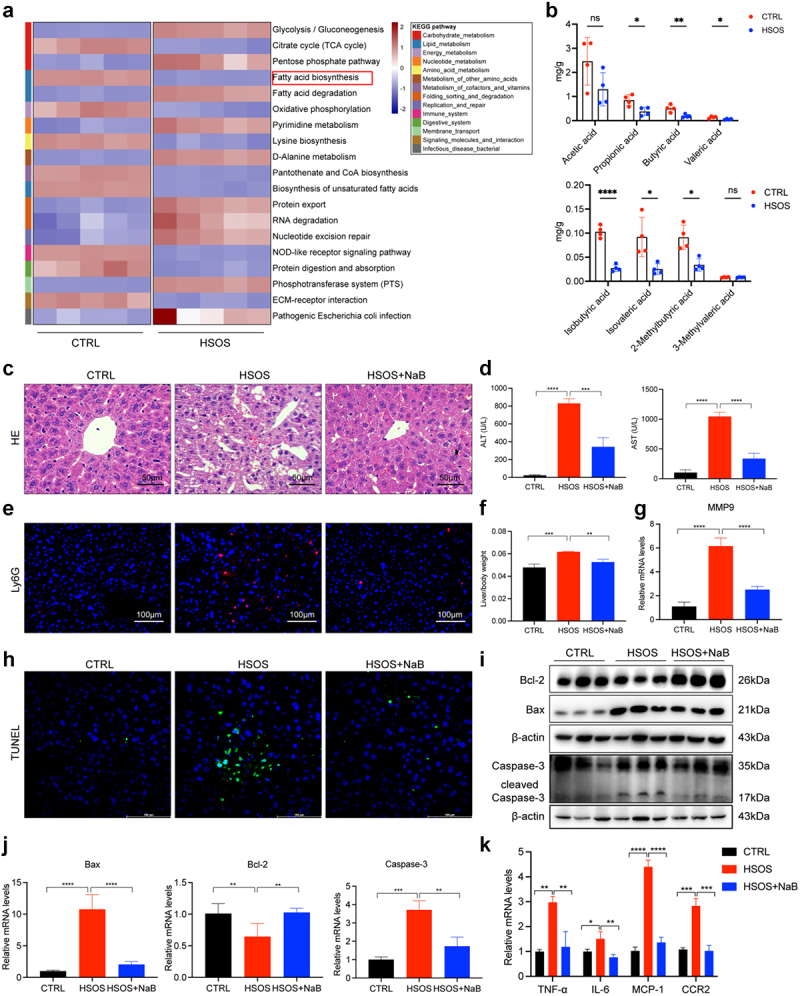
Microbiota-derived butyrate gavage delays HSOS progression. (a) Annotation of microbial gene function of CTRL and HSOS mice on KEGG pathway analysis. (b) The concentrations of SCFAs in fecal samples of HSOS mice and controls were determined by GC-MS. (c) Representative images of liver tissues stained by H&E in the three groups. (d) Serum ALT and AST levels in the three groups. (e) Representative immunostaining of Ly6G in the liver tissues. (f) Liver/body weight in mice treated with MCT and butyrate. (g) The mRNA level of MMP9 in groups. (h) Necrosis induced DNA fragmentation in mouse livers was measured by TUNEL assay in the three groups. (i, j) the protein and mRNA levels of Bcl-2, Bax, and Caspase-3 were evaluated in the liver tissues. (k) The mRNA expression of proinflammatory factors (TNF-α, IL-6, MCP-1, CCR2) on liver tissue of the three groups. *N* = 4–6 per group. Data are expressed as mean ± SD. ns, no significance, *p < 0.05, **p < 0.01, ***p < 0.001, ****p < 0.0001, ****p < 0.0001 as indicated. HSOS, hepatic sinusoidal obstruction syndrome; CTRL, control; NaB, sodium butyrate; SCFAs, short-chain fatty acids.

To explore the specific mechanism by which the gut microbiota contributes to PA-HSOS development, we focused on the gut metabolites that serve as important bridges for the gut microbiota in regulating disease progression. As shown above, PA-HSOS patients were found to have low levels of beneficial bacteria (mainly bacteria producing SCFAs). Kyoto Encyclopedia of Genes and Genomes (KEGG) enrichment showed that the fatty acid biosynthesis pathway was remarkably downregulated in HSOS mice compared with CTRL mice (*p* < 0.001) (Figure 3A). It is well known that SCFAs are produced by intestinal microbial fermentation of indigestible dietary fiber, which are one of the most studied categories of metabolites involved in host-microbiota interactions. Therefore, SCFAs levels from CTRL and HSOS mice were further quantified. A statistically relevant decrease in most SCFAs was observed compared with the control group (Figure 3B). The mean relative contents of fecal SCFAs and *p* values were presented in Supplementary Table S4 between the two groups. It has been reported that acetic acid, propionic acid, and butyric acid have the dominant content among SCFAs, accounting for more than 85%,^31,32^ which is consistent with our findings that these three substances constituted most of the SCFAs contents. Then, we focused on the three SCFAs. Of these, butyrate, with the most significant difference, was selected for analysis, which has also been shown to play an important role in modulating inflammatory response and intestinal barrier functions.^33,34^ Next, to further evaluate the effect of butyrate on intestinal injury and the progression of HSOS in vivo, we administered butyrate to drinking water prior to MCT induction. Notably, butyrate supplementation markedly alleviated hepatomegaly and liver congestion, decreased liver/body weight and serum biochemical parameters, reduced MMP9, and diminished infiltrated neutrophils (Figure 3C-G). In line with improved liver architecture, butyrate-treated HSOS mice demonstrated fewer TUNEL positive cells than HSOS mice (Figure 3H). Consistently, as revealed by western blot analysis, apoptosis-related Bax and cleaved-Caspase-3 were reduced and the anti-apoptotic Bcl-2 was significantly improved by butyrate (Figure 3I). Further confirmation of this observation was also obtained by PCR analysis (Figure 3J). In addition, significant variations with butyrate supplementation were noted for hepatic inflammatory cytokines, such as TNF-α, IL-6, MCP-1, and CCR2 (Figure 3K). Subsequently, to further clarify whether butyrate could also inhibit hepatocyte apoptosis in vitro, flow cytometric analysis was performed in an MCT and butyrate cocultured LO2 cell model. Surprisingly, as shown in Figure S5, the apoptotic fraction was not significantly altered by continuous exposure to butyrate, regardless of the concentration, which suggested that butyrate suppressed PA-HSOS progression not by direct exposure to hepatocytes but via other mechanisms.

### Butyrate relieves intestinal barrier injury in mice with PA-HSOS

**Figure 4.**
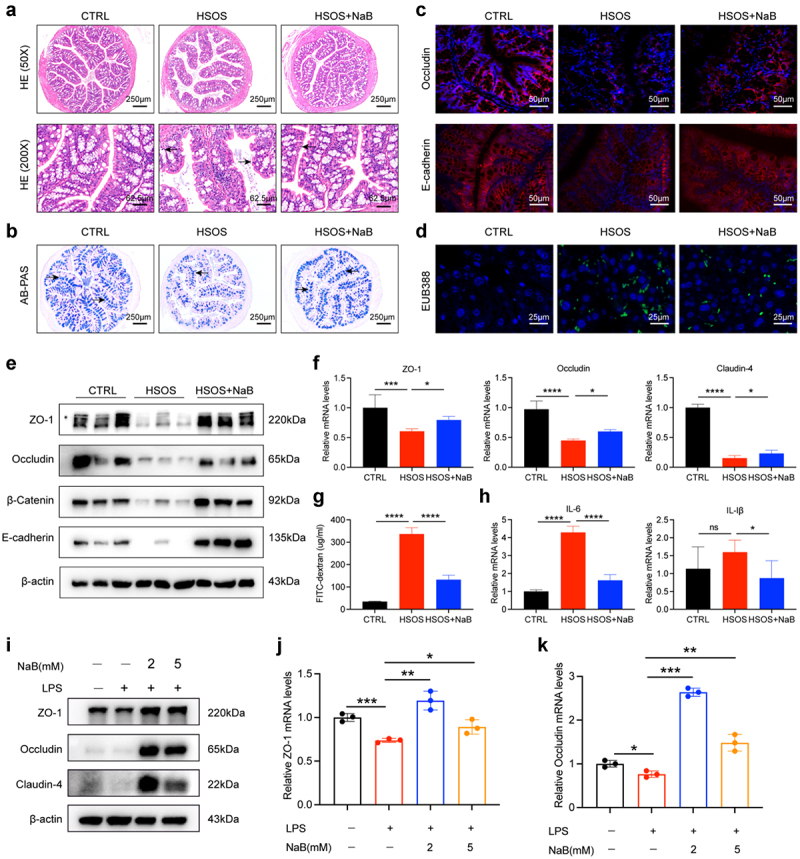
Butyrate relieves intestinal barrier injury in mice with PA-HSOS. (a) H&E staining of colon sections from individuals with or without butyrate. Black arrow indicated inflammatory cells infiltration. (b) AB-PAS staining of colon sections from individuals with or without butyrate. Black arrow indicated representative goblet cells. (c) Representative immunostaining of Occludin and E-cadherin in colon tissues. (d) Representative immunostaining of EUB388 in colon tissues by FISH. (e) Protein expression of ZO-1, Occludin, E-cadherin, and β-catenin in the colon. (f) Relative mRNA expression of ZO-1, Occludin and Claudin-4 in the colon. (g) Intestinal leakage measured by FITC-Dextran concentration in serum. (h) The mRNA expression of proinflammatory factors (IL-6, IL-1β) on colon tissue of the three groups. (i) Relative protein expression of ZO-1, Occludin, and Claudin-4 in NCM460 cells stimulated with LPS (5ug/ml) for 24 h. (j, k) Relative mRNA expression of ZO-1(j) and Occludin (k) in NCM460 cells stimulated with LPS (5ug/ml) for 24 h. *N* = 6 per group. Data are expressed as mean ± SD. ns, no significance, *p < 0.05, **p < 0.01, ***p < 0.001, ****p < 0.0001 as indicated. HSOS, hepatic sinusoidal obstruction syndrome; CTRL, control; LPS, lipopolysaccharides; NaB, sodium butyrate.

Butyrate, which is rapidly absorbed by the intestinal mucosa and acts as an energy source, is known to be involved in intestinal barrier integrity. We therefore assessed the effect of butyrate on the intestinal barrier, permeability and inflammation in butyrate-treated HSOS mice and control HSOS mice. It was observed that HSOS mice indicated infiltration of inflammatory cells in the HE-stained micrographs (Figure 4A), and treatment with butyrate resulted in a significantly higher number of AB-PAS-stained cells in colon tissue (Figure 4B). Consistent with the PCR and immunofluorescence analysis, HSOS mice exhibited a downregulated level of tight junction or adherens junction proteins, including ZO-1, Occludin, E-cadherin, and β-catenin, and these changes were mitigated in butyrate-treated HSOS mice (Figure 4C, E, F). A FISH assay was also performed, and the results showed that butyrate treatment led to a dramatic reduction in FISH-positive bacteria (Figure 4D). Simultaneously, compared to the control groups, butyrate-pretreated mice displayed obviously mitigated gut permeability (reflected by decreased diffusion of 4 KDa FITC-dextran into the blood), indicating HSOS-induced intestinal damage (Figure 4G). Furthermore, the mRNA levels of IL-6 and IL-1β were also measured in mouse colon tissue, and the results exhibited an improved inflammatory microenvironment in HSOS mice treated with butyrate (Figure 4H). A similar expression pattern of ZO-1 and Occludin was observed in an LPS and butyrate cocultured NCM460 cell model at both the protein and mRNA levels (Figure 4I-K). From the above comprehensive analysis, these data implied that butyrate feeding significantly alleviated the HSOS disease progression through improving gut integrity. Nevertheless, the specific molecular mechanism by which butyrate contributes to intestinal barrier protection in PA-HSOS requires further investigation.

### Gut microbiota alters intestinal barrier function to reduce M1 liver macrophage polarization in a butyrate-dependent way in PA-HSOS

**Figure 5.**
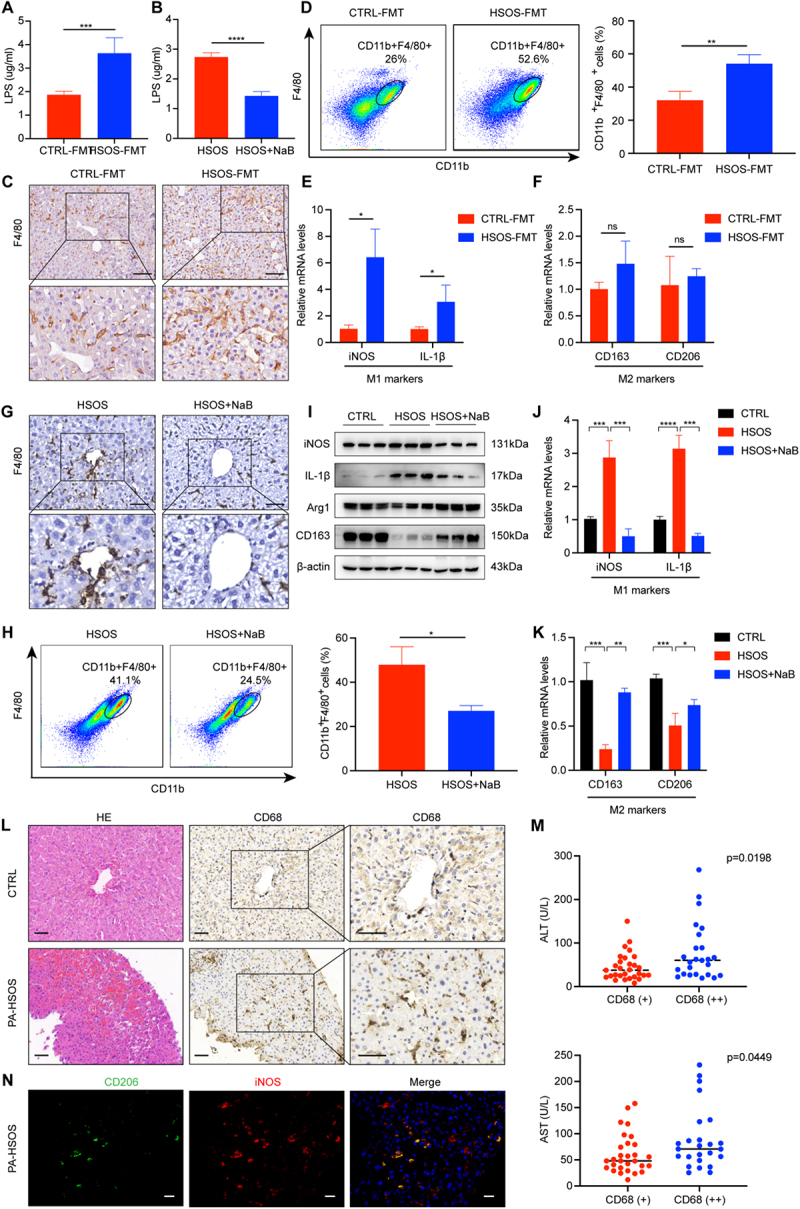
Gut microbiota alters the intestinal barrier function to reduce M1 macrophage polarization in a butyrate-dependent way in PA-HSOS. (a) LPS concentrations in the portal vein of CTRL-FMT and HSOS-FMT mice. (b) LPS concentrations in the portal vein of HSOS and butyrate treatment mice. (c) Representative immunohistochemistry images of F4/80 in CTRL-FMT and HSOS-FMT mice liver tissues. (d) Representative plots of flow cytometry and bar plots of macrophages in liver nonparenchymal cells. (e, f) Relative expression of M1 macrophages (iNOS, IL-1β) and M2 macrophages (CD163, CD206). (g) Representative immunohistochemistry images of F4/80 in HSOS and butyrate treatment mice liver tissues. (h) Representative plots of flow cytometry and bar plots of macrophages in liver nonparenchymal cells with or without butyrate. (i) The protein expression of iNOS, IL-1β, Arg-1, and CD163 in the liver. (j, k) Relative expression of M1 macrophages (iNOS, IL-1β) and M2 macrophages (CD163, CD206) in mice with or without butyrate. (l) Representative H&E staining and macrophages CD68 staining of liver tissue in control (*n* = 6) and PA-HSOS patients (*n* = 6). (m) Correlation analysis between CD68 and ALT, AST in patients with PA-HSOS (*n* = 54). (n) Double-immunofluorescence staining for CD206 and iNOS in liver tissues of patients with PA-HSOS. *N* = 3–6 per group. Data are expressed as mean ± SD. Scale bars in images represent 150 μm. ns, no significance, *p < 0.05, **p < 0.01, ***p < 0.001, ****p < 0.0001 as indicated. HSOS, hepatic sinusoidal obstruction syndrome; CTRL, control; LPS, lipopolysaccharides; NaB, sodium butyrate; FMT, fecal microbiota transplantation.

Liver macrophages, including resident macrophages and infiltrated macrophages, play an essential role in the immune regulation of liver homeostasis.^12^ Hence, considering the alleviation of intestinal barrier function through regulating gut microbiota and supplementing butyrate, we next explored whether they had an effect on liver macrophage polarization. It is known that intestinal injury can promote significant absorption of LPS into the portal circulation, which is a classical inflammatory inducer of macrophages, promoting polarization to the M1 phenotype.^35^ We first detected the concentration of serum LPS in each group, and the results previously mentioned suggested that the plasma concentration of LPS in the HSOS mice was significantly higher than that in the control group (Figure S3C). Notably, the LPS concentration in the portal vein was dramatically decreased in CTRL-FMT-treated and butyrate-treated HSOS mice (Figure 5A, B). Afterward, we assessed macrophage infiltration and activation in the liver. As observed in Figure 5C, D, macrophage infiltration was greatly increased in HSOS-FMT mice in both immunohistochemistry and flow cytometric analysis. At the same time, a high level of expression of M1 markers was detected in our PCR results, pointing to infiltrated macrophages as M1 macrophages (Figure 5E, F). The same change could be found in the butyrate-treated mice (Figure 5G-K). This phenomenon could be reversed after butyrate supplementation. We also assessed the infiltration and activation status of macrophages in the liver tissues of PA-HSOS patients. Compared to normal liver tissues, PA-HSOS tissues showed significantly higher levels of macrophage marker CD68 (Figure 5L), and it was positively correlated with ALT (*p* = 0.0198) and AST (*p* = 0.0449) elevation (Figure 5M). Moreover, the M1/M2 phenotype of macrophages was further characterized by using iNOS (M1) and CD206 (M2). As we expected, PA-HSOS patients were predominantly infected with inflammatory macrophages (M1) (Figure 5N). These results suggested that the gut microbiota and its metabolite butyrate alleviated PA-HSOS by reducing macrophage M1 polarization through regulating intestinal barrier function.

### Depletion of liver macrophages ameliorates liver injury induced by MCT

**Figure 6.**
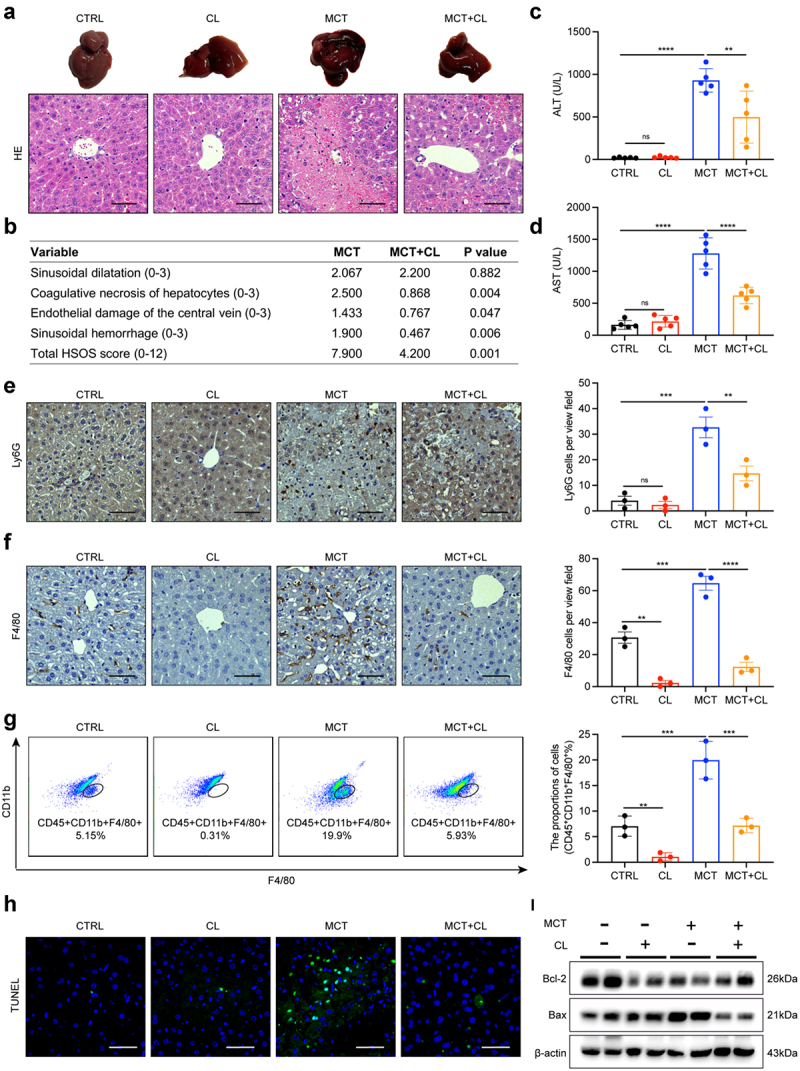
Depletion of liver macrophages ameliorates liver injury induced by MCT. (a) Representative macroscopic views of liver and HE staining images of liver tissues in four groups. (b) HSOS scores for H&E staining in a mouse model of MCT-induced HSOS. (c, d) Serum ALT (c) and AST (d) levels in four groups. (e) Representative neutrophils Ly6G staining images of liver tissues in four groups. (f) Representative macrophages F4/80 staining images of liver tissues in four groups. (g) Representative flow cytometry plots and percentage of CD45^+^CD11b^+^F4/80^+^ macrophages in NPCs from liver of the four groups. (h) Necrosis induced DNA fragmentation in mouse livers was measured by TUNEL assay in four groups. (i) The protein levels of Bcl-2 and Bax in liver tissues were determined by western blot. *N* = 3–5 per group. Data are expressed as mean ± SD. Scale bars in images represent 50 μm. ns, no significance, *p < 0.05, **p < 0.01, ***p < 0.001, ****p < 0.0001 as indicated. HSOS, hepatic sinusoidal obstruction syndrome; CTRL, control; MCT, monocrotaline; CL, clodronate liposomes; NPCs, nonparenchymal cells.

We have previously demonstrated the infiltration and activation of macrophages initiated by intestinal damage in PA-HSOS patients and mice. To further explore the role of macrophage activation in vivo, we used clodronate liposomes for the clearance of macrophages in mice and then evaluated their HSOS progression. As expected, the gross appearance of the liver significantly improved compared to that of the control group (Figure 6A). HE staining results showed that there were apparent changes in sinusoidal hemorrhage and hepatocellular necrosis, except hepatic sinusoidal dilatation (Figure 6A). This was accompanied by a lower histologic score, reduced serum ALT and AST, and lower levels of Ly6G (Figure 6B-E). Successful macrophage depletion was also confirmed by F4/80 immunohistochemical staining (Figure 6F) and flow cytometric analysis (Figure 6G). We next investigated the levels of apoptotic cells in each group by TUNEL and WB analysis. Pro-apoptotic Bax was decreased, while anti-apoptotic Bcl-2 was increased compared with that in MCT-induced HSOS mice (Figure 6H, I). We also assessed the impact of clodronate liposomes on intestinal barrier function, and no clear trend in HE and tight junction protein expression was evident, suggesting that clodronate liposome alone did not harm the intestinal (Figure S6). These data demonstrated that macrophages are involved in the progression of HSOS.

## Discussion

**Figure 7.**
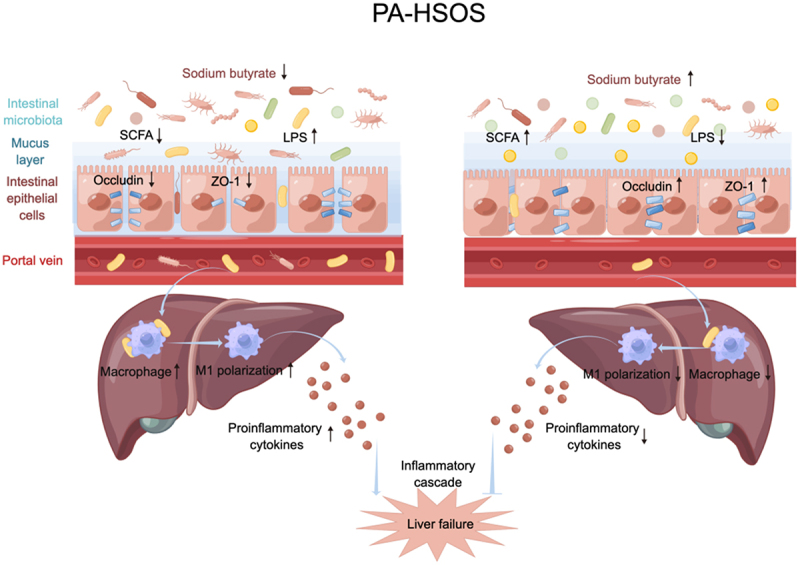
Gut microbiota promotes macrophages M1 polarization in HSOS via regulating intestinal barrier function mediated by butyrate. Gut flora disorders in HSOS contributes to a decrease of butyrate in the intestine. Reduced butyrate weakens intestinal barrier function by diminishing the thickness of mucus layer and the formation of tight-junction protein (Occludin and ZO-1). Gut-derived LPS diffuses via the disrupted intestinal barrier from the intestine into the portal vein, subsequently activates liver macrophages M1 polarization and accelerates HSOS progression, ultimately results in liver failure and even multiple organ failure. These effects could be reversed by treatment with butyrate. PA-HSOS, pyrrolizidine alkaloids induced hepatic sinusoidal obstruction syndrome; SCFA, short-chain fatty acid; LPS, lipopolysaccharides.

A growing body of evidence suggests that intestinal dysbiosis is linked to the pathogenesis of several liver diseases. It has been reported that intestinal microbiota alterations influence antitumour immune surveillance and drive NAFLD progression toward cancer,^36^ while regulating the intestinal flora could improve NAFLD outcomes. As one of the most serious liver injuries, there has been increasing attention to the relationship between PA-HSOS and the gut microbiota. Although a recent study implicated the gut microbiota in the development of PA-HSOS, it has not yet been confirmed in human subjects, and the mechanism of gut microbes regulating PA-HSOS remains unclear.^19^ Herein, we demonstrated that PA-HSOS could induce dysfunction of the intestinal tight junction barrier, which is highly related to dysbiosis of the gut microbiota and SCFAs. Increased intestinal permeability promotes the release of bacterial LPS into the liver through the portal vein, which can accelerate M1 macrophage polarization and inflammatory cytokine secretion and in turn exacerbate liver damage (Figure 7). Our results might offer novel insights for the development of individualized therapeutic targets for PA-HSOS patients.

Our findings showed that PA-HSOS was associated with gut dysbacteriosis in both humans and mice. Compared to healthy controls, the major feature of intestinal microbial dysbiosis in PA-HSOS was the decrease in beneficial and SCFA-producing bacteria and the overabundance of potential pathogenic bacteria. At the phylum level, the dominant Firmicutes declined, and Proteobacteria was increased, playing an essential role in protecting the intestinal barrier and promoting intestinal health. Regarding the families of bacteria, the abundance of Lachnospiraceae and Acidaminococcaceae decreased significantly in HSOS patients. Lachnospiraceae is the best-characterized microbiota constituent that has been reported to produce SCFAs exerting a beneficial effect on the intestinal barrier, especially butyrate, via the metabolism of carbohydrate pathways.^37,38^ Likewise, Acidaminococcaceae was described to participate in the production of SCFAs to maintain the morphology and function of colonic epithelial cells. Our study is also in accordance with the previous finding by Li et al. to a certain extent.^19^ They verified that retrorsine (RTS)-induced mice had different degrees of disorders in the intestinal flora. In parallel, the relative abundance of beneficial and harmful bacteria was not quite in accordance with our results. These differences probably relate to the species, drug dosage, model establishment, environmental conditions and other factors. Meanwhile, such effects have not been confirmed in humans, and studies are warranted to investigate the in-depth mechanisms for how the gut microbiota affects intestinal inflammation and disease progression in PA-HSOS. Therefore, we further performed FMT in HSOS mice. Since fecal samples from patients for FMT were characterized by large individual differences, time-consuming and low repetition rate, and the fecal samples mostly were collected by the patient, factors regarding collection were difficult to standardize, which may lead to the instability of the final analysis results. With this in mind, we used fecal samples from mice as a donor for our FMT studies, and the concordant results suggested that HSOS-FMT exhibited intestinal injury and aggravated the progression of PA-HSOS compared with that in CTRL-FMT mice. It is implicit that dysregulation of the intestinal flora is likely an important mechanism for the development and progression of HSOS.

According to previous reports, gut microbiota disturbance has a profound impact on the development of liver injury and inflammation, highlighting the critical role of the gut – liver axis, which was focused on HSOS by Lin et al. .^18,39^ This is the first study to elaborate that trace levels of alkaloid components in bile might disrupt the intestinal mucosal barrier directly. This impairment may explain the observed inflammation, but clinically, intestinal damage is more widespread and severe. Liver-derived reactive PA metabolites prevent us from rationalizing a plausible explanation for this disparity, perhaps just as a trigger. A growing number of studies are supportive of the idea that subdiaphragmatic vagotomy (SDV) plays a key role in regulating the normal function of the gut microbiota – liver – brain as well as having important effects on insulin resistance, systemic inflammation and behavioral phenotypes like depression, decreased appetite, and vomiting.^40–42^ Interestingly, previous literature mentioned that some tryptophan-metabolizing bacteria reduced in MCT-treated rats, such as Bacteroides, Lactobacillus and Clostridium, and accompanied by a decrease in microbial tryptophan metabolic activity, which was critical in regulating pain, anxiety, cognitive impairments, and depression-related behaviors.^43^ We guessed whether HSOS mice had depression-related behaviors similar to those with other liver diseases, including hepatic ischemia/reperfusion injury, alcoholic liver disease, and nonalcoholic fatty liver disease.^44,45^ However, there are no reports showing depression-like phenotypes in HSOS. There are also no reports on the role of the gut – liver – brain axis, via the vagus nerve, in rodents with HSOS. Further study on the role of SDV on depression-like behaviors in HSOS is needed. Besides, the gut microbiota has recently gained attention as a therapeutic target for maintenance of the biological barrier of the intestine.^46^ In our experiment, we found that the transfer of microbiota from HSOS mice to WT recipient mice exacerbated HSOS after ABX treatment. We wondered whether there are other elements involved in the intestinal barrier function and progression of PA-HSOS.

Numerous previous reports in the literature have shown that most SCFAs in the human body, including acetic acid, propionic acid, butyric acid, isovaleric acid, valeric acid, and isovaleric acid, are derived from fermentation of nondigestible carbohydrates by the resident bacteria in the gastrointestinal tract, among which butyric acid is one of the main SCFAs with higher relative abundance.^47^ Within our study, HSOS mice had a significantly lower fecal butyric acid concentration than the control. It has been well documented that butyrate, an energetic metabolite, can maintain colonic epithelium homeostasis and promote tight junction (TJ) protein expression, and a deficiency in butyrate is often accompanied by pathological features, including neutrophil infiltration, which triggers inflammatory reactions in colon tissue and results in various gut-related diseases.^48,49^ The beneficial effects of butyrate on intestinal epithelial function occur through multiple mechanisms, including inducing cell differentiation, stimulating the expression of mucins such as the dominant mucin-2, and promoting crypt intestinal epithelial cell (IEC) differentiation.^50^ Apart from gut absorption, butyric acid was also reported to play a key role at the systemic level.^51^ Zhao et al. indicated that butyrate could attenuate hepatic steatosis and improve the lipid profile by activating the LKB1-AMPK-Insig signaling pathway.^52^ However, we did not find an antiapoptotic effect on LO2 cells in vitro in our preliminary experiment. In this setting, the protective role of butyrate on liver cells differed greatly depending on the drug intervention and cell type. Moreover, the majority of luminal butyrate is consumed in the gut, where it exerts its main functions, resulting in a relatively low concentration of butyrate in the portal vein.^53–55^ Therefore, this may explain why butyrate acts primarily on the intestine but not the liver in PA-HSOS. Certainly, this is worthy of further study to elucidate the mechanism responsible for the protective effects against the gut.

As the main pathogenic component of intestinal bacteria, LPS is extremely important in triggering the immune response in the host.^56^ It is primarily through the intestinal barrier that LPS can enter systemic circulation. In the present study, impaired intestinal barrier and elevated LPS serum levels were detected in humans and mice with PA-HSOS. Upon stimulation with LPS in the portal vein, liver macrophages can be polarized to classical M1 macrophages, causing uncontrolled release of inflammatory cytokines and oxidative stress and eventually resulting in liver failure.^57^ Moreover, macrophages play a substantial role in the pathogenesis and development of liver injury mediated by toxins, chemical substances, and pharmacological agents.^58^ Depletion of macrophages or inhibition of macrophage infiltration prevents liver injury and mortality.^59^ In line with these studies, we also found that intragastric administration of MCT induced a more severe inflammatory response characterized by M1 macrophage infiltration in HSOS mice than in WT mice, whereas removing macrophages reduced hepatocyte apoptosis and liver damage. Furthermore, increased M1 macrophage infiltration was reversed in FMT and butyrate supplementation experiments. These data indicated that the gut microbiota altered the intestinal barrier function to reduce M1 macrophage polarization in a butyrate-dependent manner.

Regarding the limitations of this study. First, 18 patients who underwent 16S rDNA sequencing were enrolled in our study, which may not well represent the change in the gut microbiota of the whole patient. In addition, FMT and butyrate treatment have only been evaluated in the murine model, and whether this effect occurring in PA-HSOS patients is unknown. Thus, clinical data needs to be further accumulated to provide theoretical basis for clinical application. Finally, although we can demonstrate a correlation with gut microbiota dysbiosis and the progression of existing HSOS, we cannot prove causality. Our work is gradually in progress to address these problems, and the relationship between intestinal flora and PA-HSOS remains to be further explored.

## Conclusions

Our study demonstrated that PA-HSOS was accompanied by impaired intestinal function, decreased microbiota diversity and changes in bacterial-derived factors. Disordered gut microbiota could alter intestinal barrier function to reduce M1 macrophage polarization in a butyrate-dependent manner. These results established a close interaction between the gut microbiota and MCT-induced liver injury through butyrate, thereby providing novel insights into the underlying mechanisms of liver injury induced by MCT. The modulation of intestinal microbiota or its metabolites, especially those targeting butyrate, may be a promising therapy in PA-HSOS.

## Supplementary Material

supplementary_manuscript clean.docx

## Data Availability

The data underlying this article will be shared on reasonable request to the corresponding author.

## List of abbreviations

PA-HSOS, pyrrolizidine alkaloids induced hepatic sinusoidal obstruction syndrome; MCT, monocrotaline; 16S rDNA, 16S ribosomal DNA; FMT, fecal microbiota transplantation; HVOD, hepatic venous occlusive disease; SECs, sinusoidal endothelial cells; HSCT, hematopoietic stem cell transplantation; GSH, glutathione; TIPS, transjugular intrahepatic portosystemic shunt; ROS, reactive oxygen species; ALT: alanine aminotransferase; AST: aspartate aminotransferase; MMP9: matrix metalloproteinase 9; TLR4, toll-like receptor 4; NAFLD, non-alcoholic fatty liver disease; HCC, hepatocellular carcinoma; BT, bacterial translocation; SCFAs, short-chain fatty acids; AB-PAS, Alcian blue-Periodic acid Schiff; LPS, lipopolysaccharides; OTU, operational taxonomic unit; KEGG, Kyoto Encyclopedia of Genes and Genomes; RTS, retrorsine; IECs, intestinal epithelial cells; NPCs, nonparenchymal cells; SDV, subdiaphragmatic vagotomy.
