# Supplementary material for: Gut microbiota promotes macrophage M1 polarization in hepatic sinusoidal obstruction syndrome via regulating intestinal barrier function mediated by butyrate
Source: Gut Microbes. 2024 Jul 16;16(1):2377567. doi: 10.1080/19490976.2024.2377567 (PMC11253885; doi:10.1080/19490976.2024.2377567)
Supplement: supplementary_manuscript clean.docx [file KGMI_A_2377567_SM4716.docx]

**Gut microbiota promotes macrophages M1 polarization in hepatic sinusoidal obstruction syndrome via regulating intestinal barrier function mediated by butyrate**

**Supplementary materials:**

***A table of contents***

***1. Supplemental Figures (6)***

***2. Supplemental Tables (4)***


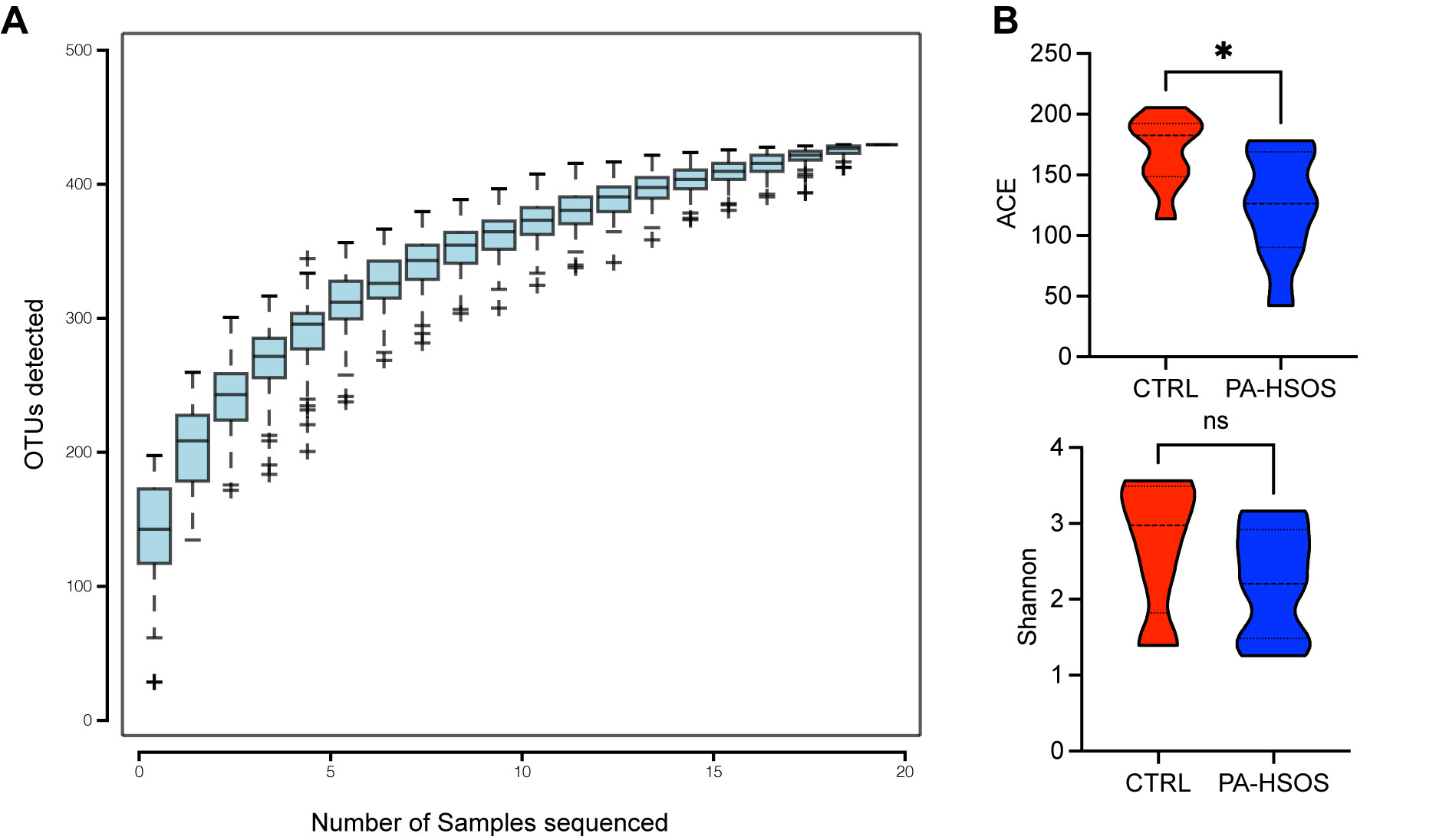


**FIGURE S1** Sequencing depth and diversity of microflora in the healthy and HSOS patients. (A) The sequencing depth detected by 16S rRNA in patients (n=9 per group). (B) ACE and Shannon diversity indexes of the gut microbiota between the two group. ns, no significance, *p< 0.05 as indicated. HSOS, hepatic sinusoidal obstruction syndrome; CTRL, control.


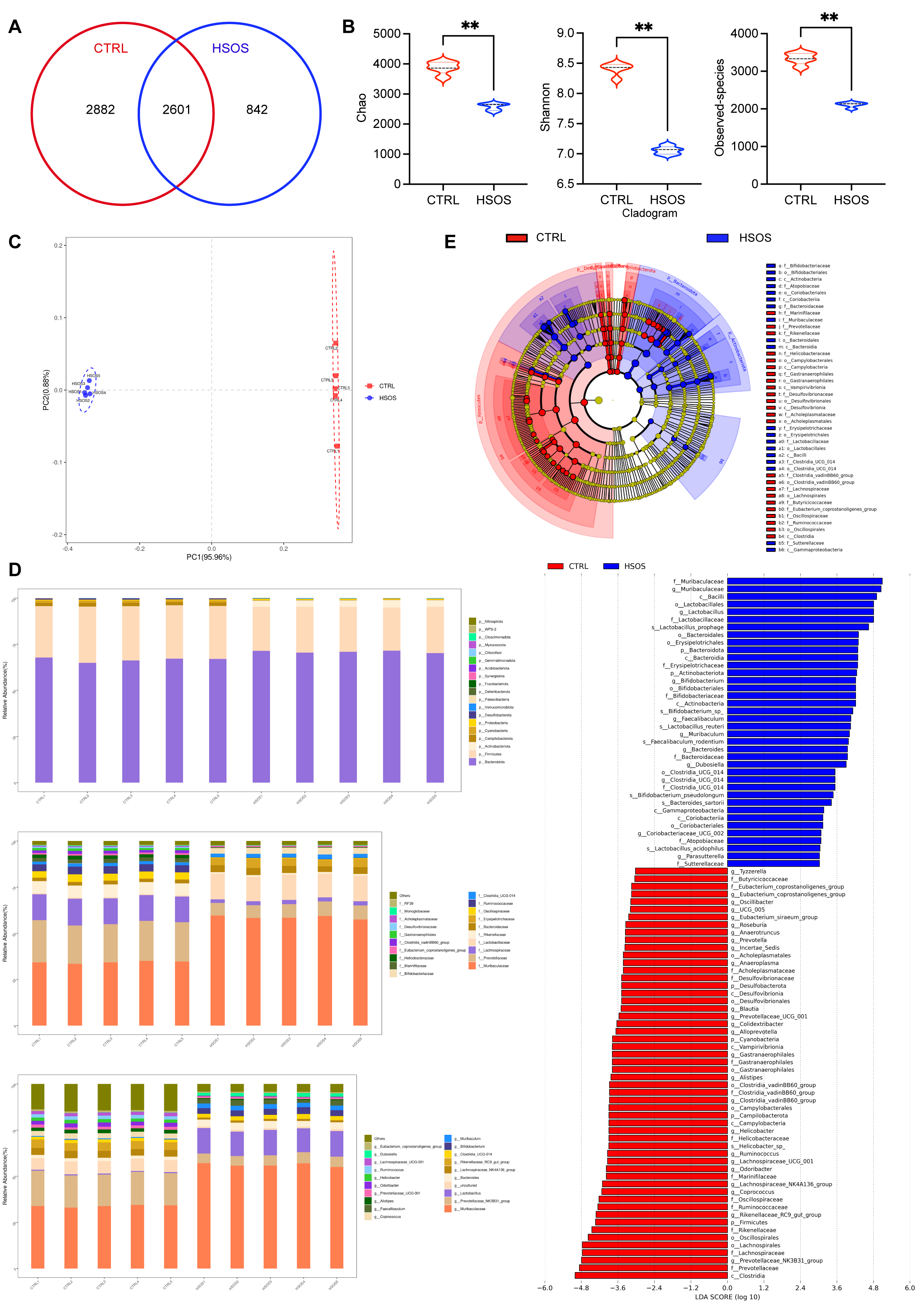


**FIGURE S2** HSOS mice exhibits gut bacterial microbiota dysbiosis. (A) In genus level, venn diagram of the OTUs in the CTRL and PA-HSOS mice. (B) Chao, Shannon, and Observed-species diversity indexes of the gut microbiota between the two groups. (C) Principal component analysis (PCA) plot of similarities among the two groups. (D-F) Bar charts of the gut microbiota composition at the phylum (D), family (E), and genus (F) levels in CTRL group and HSOS group. (E) Cladogram generated from linear discriminant analysis effect size (LEfSe) and the LDA score. N=5 per group. **p<0 .01 as indicated. HSOS, hepatic sinusoidal obstruction syndrome; CTRL, control.


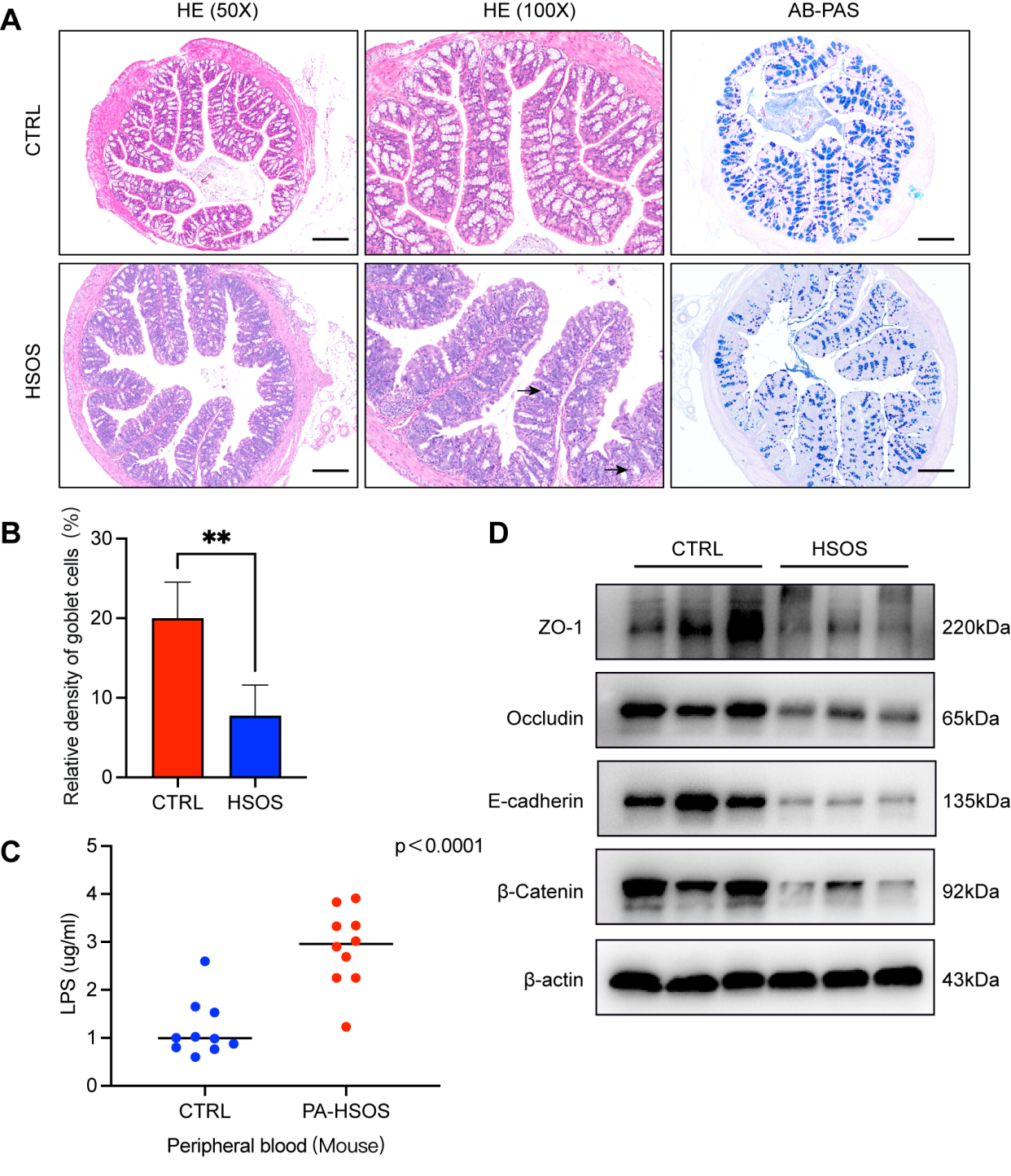


**FIGURE S3** HSOS mice exhibits impaired gut barrier. (A) H&E staining and AB-PAS staining of colon sections in HSOS mice and the control. Black arrow indicated inflammatory cells infiltration. (B) The bar graph of goblet cells by AB-PAS. (C) The plasma concentrations of LPS in CTRL group (n=10) and HSOS group (n=10) (t=5.315). (D) Protein expression of ZO-1, Occludin, E-cadherin, and β-catenin in the colon. Data are expressed as mean ± SD. Scale bars in images represent 250μm. N=5 per group. **p<0 .01 as indicated. HSOS, hepatic sinusoidal obstruction syndrome; CTRL, control.


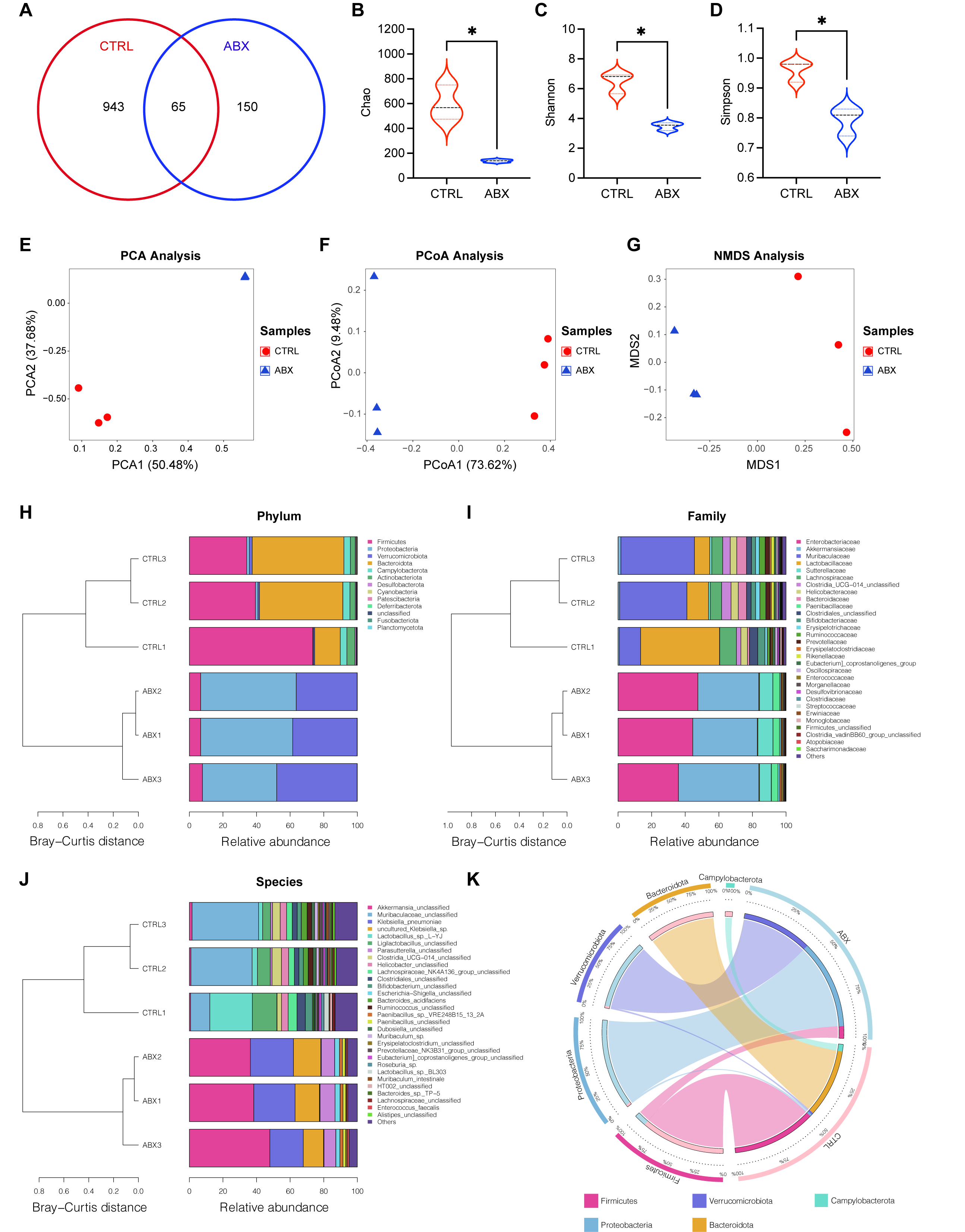


**FIGURE S4** Antibiotic cocktail treatment removes most intestinal flora in mice. (A) Venn diagram of the OTUs in the CTRL and ABX mice. (B-D) Chao, Shannon, and Simpson diversity indexes of the gut microbiota between the two groups. (E-G) Principal component analysis (PCA), Principal coordinate analysis (PCoA), and Nonmetric Multidimensional Scaling (NMDS) plot of similarities among the different groups. (H-J) Bar charts of the gut microbiota composition at the phylum, family, and species levels in CTRL group and ABX group. (K) A Circos analysis of distribution and proportion of the microbial dominant genera at the phylum level. N=3 per group. Data are expressed as mean ± SD. *p< 0.05 as indicated. CTRL, control; ABX, antibiotic cocktail.


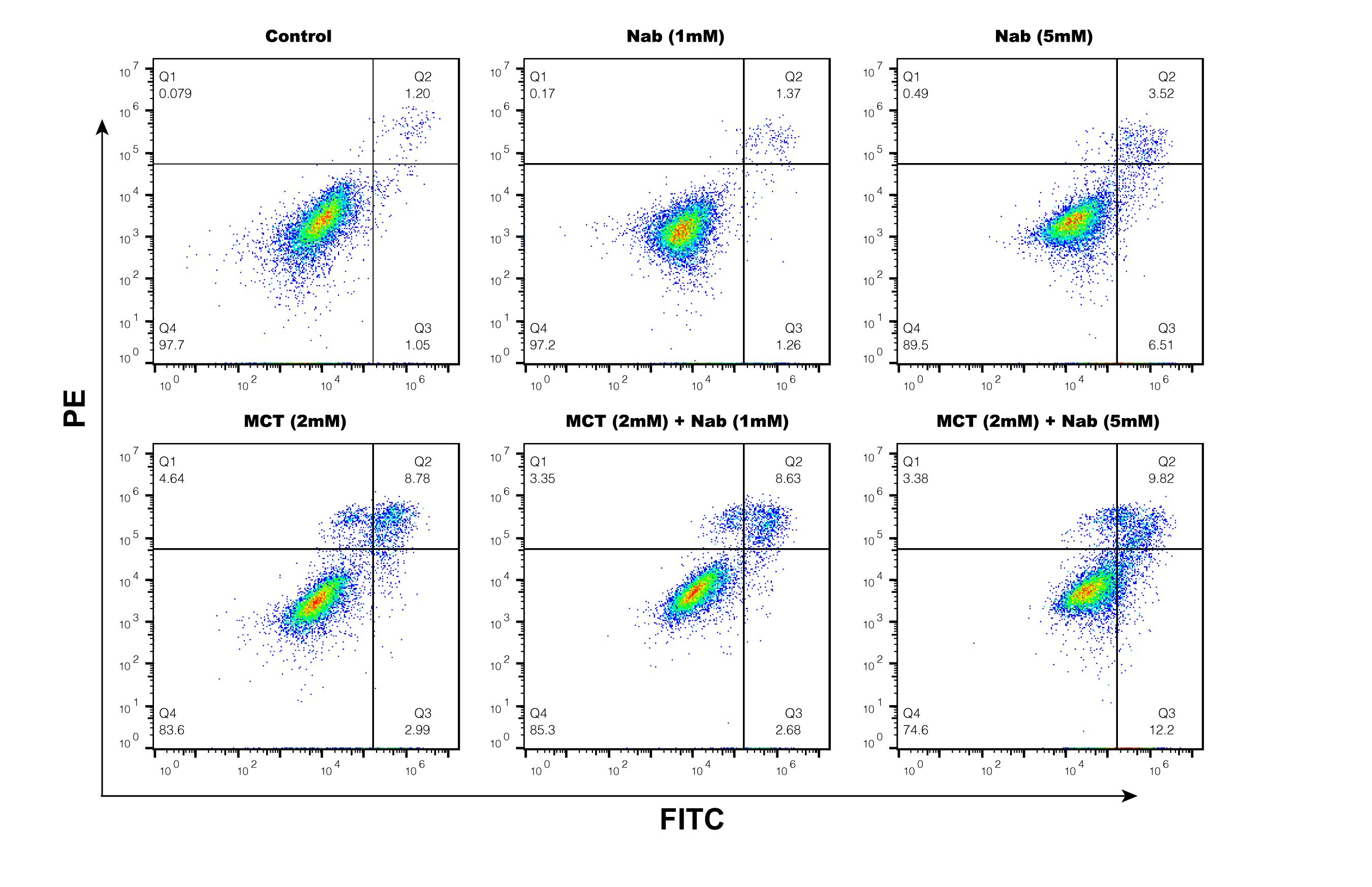


**FIGURE S5** Butyrate does not improve the hepatocyte apoptosis induced by MCT. Representative apoptosis plots of flow cytometry in LO2 co-incubated with MCT (2mM) or NaB for 24h. MCT, monocrotaline; NaB, sodium butyrate.


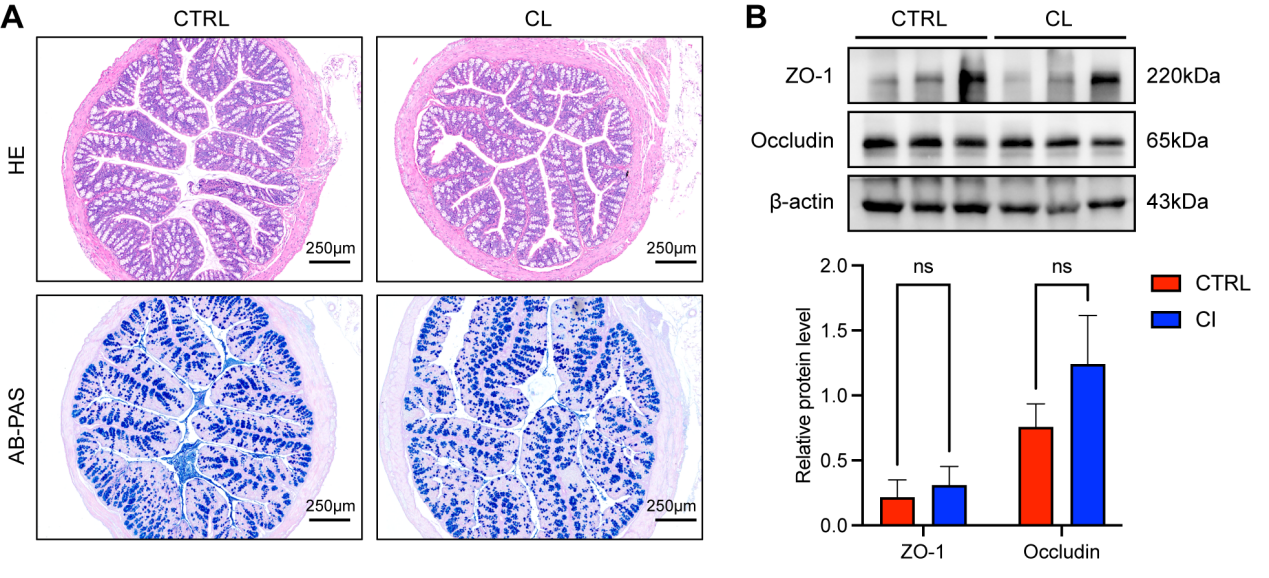


**FIGURE S6** Clodronate liposomes had no effect on gut barrier. (A) H&E staining and AB-PAS staining of colon sections in the CTRL and CL mice. (B) Protein expression of ZO-1 (t=0.477, p=0.659) and Occludin (t=1.167, p=0.308) in the colon. Data are expressed as mean ± SD. Scale bars in images represent 250μm. N=5 per group. ns, no significance. CTRL, control; CL, clodronate liposomes.

**Table S1. Clinical and laboratory characteristics of HSOS cases and controls**

| **Characteristics** | **HSOS patient (n=9)** | **Healthy control (n=9)** |
| --- | --- | --- |
| Gender |  |  |
| Male | 5 | 5 |
| Female | 4 | 4 |
| Age (year) | 63±8.03 | 61.56±10.00 |
| Hb (g/L) | 138.1±13.70 | 141.7±13.57 |
| WBC (×109/L) | 8.11±3.97 | 5.1±088 |
| PLT (×109/L) | 123.4±43.59 | 179.7±37.19 |
| ALT (U/L) | 101.2±60.52 | 18.06±7.70 |
| AST (U/L) | 121.8±59.09 | 21.56±4.89 |
| ALB (g/L) | 33.32±3.08 | 41.52±2.27 |
| TB (μmol/L) | 42.37±18.48 | 11.30±2.59 |
| PT (s) | 18.30±2.60 | 11.03±0.54 |
| APTT (s) | 35.49±5.52 | 27.30±1.64 |
| Ascites Depth (cm) | 7.81±2.80 | / |
| Peak PVV (cm/s) | 12.51±3.62 | / |
| Medication |  |  |
| antibiotic | 0 | 0 |
| low molecular weight heparin | 2 | 0 |
| hepatoprotective drug | 2 | 0 |
| diuretic | 1 | 0 |

Data are mean (SD). HSOS, hepatic sinusoidal obstruction syndrome; Hb, hemoglobin; WBC, white blood cell; PLT, platelets; ALT, glutathione aminotransferase; AST, aspartate aminotransferase; ALB, albumin; TB, total bilirubin; PT, prothrombin time; APTT, activated partial thromboplastin time; PVV, portal vein velocity.

**Table S2. Primers (H, denotes human and M, denotes mice)**

| **Gene Symbol** | **Forward Primer** | **Reverse Primer** |
| --- | --- | --- |
| *H-ZO-1* | CAACATACAGTGACGCTTCACA | CACTATTGACGTTTCCCCACTC |
| *H-OCCLUDIN* | ACAAGCGGTTTTATCCAGAGTC | GTCATCCACAGGCGAAGTTAAT |
| *H-β-ACTIN* | TCACCCACACTGTGCCCATCTACGA | CAGCGGAACCGCTCATTGCCAATGG |
| *M-Tnfa* | CAGGCGGTGCCTATGTCTC | CGATCACCCCGAAGTTCAGTAG |
| *M-Il6* | CTGCAAGAGACTTCCATCCAG | CTGCAAGAGACTTCCATCCAG |
| *M-Il1b* | GAAATGCCACCTTTTGACAGTG | TGGATGCTCTCATCAGGACAG |
| *M-Tgfb* | CGCCATCTATGAGAAAACCAA | GAGTTCCACATGTTGCTCCA |
| *M-Mmp9* | GAAGTCTCAGAAGGTGGAT | GAAATAGGCTTTGTCTTGGTA |
| *M-Ccr2* | AAGGGTCACAGGATTAGGAAG | ATGGTTCAGTCACGGCATA |
| *M-Mcp1* | TTAAAAACCTGGATCGGAACCAA | GCATTAGCTTCAGATTTACGGGT |
| *M-Bax* | TGAGCGAGTGTCTCCGGCGAAT | GCACTTTAGTGCACAGGGCCTTG |
| *M-Bcl2* | TGGTGGACAACATCGCCCTGTG | GGTCGCATGCTGGGGCCATATA |
| *M-Bad* | CCAGAGTTTGAGCCGAGTGAGCA | ATAGCCCCTGCGCCTCCATGAT |
| *M-Caspase3* | TGGGACTGATGAGGAGA | ACTGGATGAACCACGAC |
| *M-Zo1* | GGGCCATCTCAACTCCTGTA | AGAAGGGCTGACGGGTAAAT |
| *M-Occludin* | ACTATGCGGAAAGAGTTGACAG | GTCATCCACACTCAAGGTCAG |
| *M-Claudin4* | TGGAGGACGAGACCGTCAA | CACGGGCACCATAATCAGCA |
| *M-Cd163* | GGTGGACACAGAATGGTTCTTC | CCAGGAGCGTTAGTGACAGC |
| *M-Cd206* | CTCTGTTCAGCTATTGGACGC | CGGAATTTCTGGGATTCAGCTTC |
| *M-inos* | TGGAGCGAGTTGTGGATTG | CCTCTTGTCTTTGACCCAGTAG |
| *M-β-Actin* | GCTACAGCTTCACCACCACAG | GGTCTTTACGGATGTCAACGTC |

**Table S3. Corresponding t-statistics with p values or F-statistics with p values in Figures**

| **Number** | **Indicators** | **Statistics** | **p value** |
| --- | --- | --- | --- |
| Figure 1H | LPS | t=4.609 | <0.0001 |
| Figure 2C | ALT | t=6.162 | 0.0035 |
|  | AST | t=2.855 | 0.0462 |
| Figure 2D | MMP9 | t=4.805 | 0.0086 |
| Figure 2E | IL-6 | t=6.179 | 0.0035 |
|  | TNF-α | t=5.114 | 0.0069 |
|  | CCR2 | t=8.111 | 0.0013 |
| Figure 2G | Bax | t=9.280 | 0.0008 |
|  | Bad | t=5.188 | 0.0066 |
|  | Bcl-2 | t=5.353 | 0.0059 |
| Figure 2K | ZO-1 | t=8.072 | 0.0013 |
|  | Occludin | t=19.37 | <0.0001 |
| Figure 2L | serum FITC-Dextran level | t=7.132 | 0.002 |
| Figure 2N | ZO-1 | F (2, 6) = 32.46 | 0.0006 |
|  | Occludin | F (2, 6) = 45.91 | 0.0002 |
| Figure 3B | acetic acid | t=1.933 | 0.1014 |
|  | propionic acid | t=3.361 | 0.0152 |
|  | butyric acid | t=3.886 | 0.0081 |
|  | isobutyric acid | t=10.44 | 0.0000 |
|  | 2-Methylbutyric acid | t=4.009 | 0.0070 |
|  | isovaleric acid | t=3.171 | 0.0193 |
|  | valeric acid | t=3.262 | 0.0172 |
|  | 3-Methylvaleric acid | t=0.3124 | 0.7653 |
| Figure 3D | ALT | F (2, 15) = 111.9 | <0.0001 |
|  | AST | F (2, 15) = 142.3 | <0.0001 |
| Figure 3F | Liver/body weight | F (2, 15) = 31.46 | 0.0007 |
| Figure 3G | MMP9 | F (2, 15) = 106.0 | <0.0001 |
| Figure 3J | Bcl-2 | F (2, 15) = 11.92 | 0.0008 |
|  | Bax | F (2, 15) = 91.31 | <0.0001 |
|  | Caspase-3 | F (2, 15) = 34.84 | 0.0005 |
| Figure 3K | TNF-α | F (2, 15) = 24.75 | 0.0013 |
|  | IL-6 | F (2, 15) = 12.45 | 0.0073 |
|  | MCP-1 | F (2, 15) = 225.9 | P<0.0001 |
|  | CCR2 | F (2, 15) = 64.50 | P<0.0001 |
| Figure 4F | ZO-1 | F (2, 15) = 13.30 | 0.0005 |
|  | Occludin | F (2, 15) = 64.98 | P<0.0001 |
|  | Claudin-4 | F (2, 15) = 512.0 | P<0.0001 |
| Figure 4G | FITC-Dextran concentration | F (2, 6) = 174.5 | P<0.0001 |
| Figure 4H | IL-6 | F (2, 15) = 246.3 | P<0.0001 |
|  | IL-1β | F (2, 15) = 3.373 | 0.0617 |
| Figure 4J | ZO-1 | F (3, 8) = 21.29 | 0.0004 |
| Figure 4K | Occludin | F (3, 8) = 148.9 | P<0.0001 |
| Figure 5A | LPS | t=5.913 | 0.0006 |
| Figure 5B | LPS | t=13.11 | <0.0001 |
| Figure 5D | macrophages | t=5.034 | 0.0073 |
| Figure 5E | iNOS | t=4.384 | 0.0118 |
|  | IL-1β | t=2.811 | 0.0483 |
| Figure 5F | CD163 | t=1.856 | 0.1370 |
|  | CD206 | t=0.511 | 0.6363 |
| Figure 5H | macrophages | t=4.269 | 0.0130 |
| Figure 5J | iNOS | F (2, 15) = 45.08 | 0.0002 |
|  | IL-1β | F (2, 15) = 98.78 | P<0.0001 |
| Figure 5K | CD163 | F (2, 15) = 36.02 | 0.0005 |
|  | CD206 | F (2, 15) = 26.04 | 0.0011 |
| Figure 6C | ALT | F (3, 16) = 34.04 | P<0.0001 |
| Figure 6D | AST | F (3, 16) = 59.30 | P<0.0001 |
| Figure 6E | Ly6G | F (3, 8) = 25.94 | 0.0002 |
| Figure 6F | F4/80 | F (3, 8) = 72.54 | P<0.0001 |
| Figure 6G | macrophages | F (3, 8) = 37.77 | P<0.0001 |

| **SCFAs** | **Mean of CTRL (ug/g)** | **Mean of HSOS (ug/g)** | **P value** | **P value summary** |
| --- | --- | --- | --- | --- |
| Acetic acid | 2459 | 1297 | 0.1014 | ns |
| Propionic acid | 848 | 377.2 | 0.0152 | * |
| Butyric acid | 507.6 | 189.6 | 0.0081 | ** |
| Valeric acid | 135 | 54.04 | 0.0172 | * |
| Isobutyric acid | 102.9 | 27.42 | 0.0000 | **** |
| Isovaleric acid | 91.99 | 25.15 | 0.0193 | * |
| 2-Methylbutyric acid | 91.29 | 34.09 | 0.0070 | * |
| 3-Methylvaleric acid | 8.228 | 8.073 | 0.7653 | ns |

**Table S4. The mean relative contents of fecal SCFAs and p values in different groups**

HSOS, hepatic sinusoidal obstruction syndrome; CTRL, control; SCFAs, short-chain fatty acids.
